# ZnTiN_2_ as an Electron-Selective, Protective Layer on Si Photocathodes

**DOI:** 10.1021/acselectrochem.4c00155

**Published:** 2025-04-22

**Authors:** Anna C. Kundmann, John S. Mangum, Mellie Lemon, Maria Kelly, Dennice M. Roberts, Melissa K. Gish, Elisa M. Miller, Emily L. Warren, Frank E. Osterloh, Ann L. Greenaway

**Affiliations:** † Department of Chemistry, 8789 University of California, Davis, California 95616, United States; ‡ Materials Chemical and Computational Science Directorate, 53405 National Renewable Energy Laboratory, Golden, Colorado 80401, United States; § Department of Chemical and Biological Engineering, 1877 University of Colorado Boulder, Boulder, Colorado 80309, United States; ∥ Renewable and Sustainable Energy Institute, 1877 University of Colorado Boulder, Boulder, Colorado 80309, United States

**Keywords:** Photoelectrochemistry, ternary nitride, protection layer, solid-state junction

## Abstract

Photoelectrochemical production of fuels requires photoelectrodes that efficiently convert sunlight to electrochemical energy by producing photovoltage and photocurrent and maintain this ability over time under a variety of pH, illumination, and applied bias conditions. Work in the photovoltaic community has demonstrated that interfaces with high charge carrier selectivity provide high photovoltages. This offers a co-design opportunity to create semiconductor photoelectrodes with contact layers that are both carrier-selective and offer protection from degradation in aqueous solutions. In this work, we explored the ternary nitride ZnTiN_2_ as an electron-selective, protective layer for Si-based photocathodes. We demonstrated that ZnTiN_2_ formed a heterojunction with p-type Si that facilitated electron movement toward the ZnTiN_2_ surface for light-driven reduction reactions. Across a variety of electrolyte conditions, ZnTiN_2_/Si produced an open circuit voltage of ca. 400 mV vs the solution potential, while bare Si produced 220–480 mV vs the solution potential depending on conditions. ZnTiN_2_ was also shown to protect Si over 72 h at open circuit in the dark in 0.1 M KHCO_3_ aqueous solution at pH 10.5, with a 2.4% loss in open circuit voltage compared to a 17% loss for unprotected Si. A protective effect was also observed under illumination during methyl viologen reduction at pH 3.5 for 21 h, with a 2.5% loss in open circuit voltage observed for ZnTiN_2_/Si compared to a 25% loss in open circuit voltage for unprotected Si under the same conditions. Elemental characterization revealed the presence of oxides on the surface of ZnTiN_2_ that are consistent with the Pourbaix diagram after photoelectrochemical operation; these oxides appeared to support durability without hindering charge carrier extraction to drive electrochemical work. This work highlights the promise of ZnTiN_2_ for durable photoelectrochemical applications.

## Graphical abstract

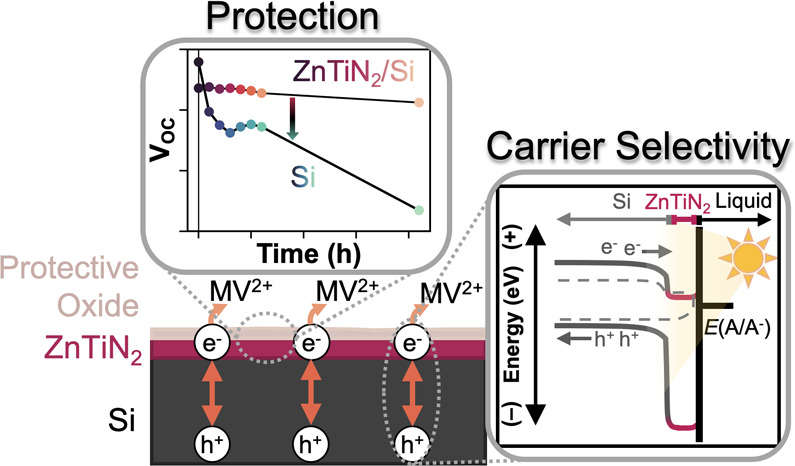

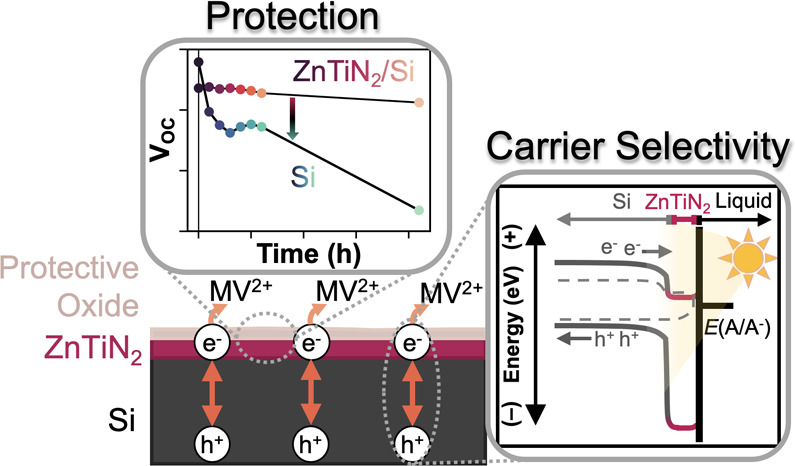

## Introduction

Photoelectrochemical (PEC) systems offer a promising route to generate fuels by using solar energy to drive endergonic reactions to form H_2_ or hydrocarbons from abundant inputs like H_2_O and CO_2_. Much like photovoltaic (PV) devices, upon illumination PEC devices must generate electrons and holes, separate them, and collect them at different contacts, thus producing high photocurrent and photovoltage. While charge carriers in PV devices are collected at solid contacts to produce current and voltage in an external circuit, charge carriers in PEC devices are collected at a liquid contact to complete the electrochemical circuit and perform (among others) fuel-forming reactions.^1^ PV research and design has benefitted from conceptualizing charge carrier separation in terms of carrier-selective contacts that allow only electrons or holes to be collected while blocking the other carrier type.^2,3^ Practically, such contacts are ideally realized via band alignment that prevents the transport of the undesired carrier type and doping that improves the conductivity of the desired carrier type. Similar strategies have begun to be adopted in PEC device designs, with benefits for device photovoltage, fill factor, and solar-to-fuel efficiency.^4–6^ As the importance of carrier-selective contacts gains greater recognition in the PEC field,^7^ there is also an opportunity for co-designing surface layers that are both carrier-selective and protective. Simultaneously meeting both of these criteria would enable devices with high efficiency while overcoming the longstanding durability challenge for PEC systems.

Silicon continues to be an attractive photoabsorber for PEC devices because it is earth-abundant,^8^ has a suitable bandgap (1.12 eV) to be a bottom absorber in a tandem PEC system,^9^ and the proliferation of Si-based PV has established much of the knowledge and infrastructure needed to make high quality absorber material and devices.^10^ Indeed, some of the best performing Si photocathodes have taken advantage of developments in the PV community regarding surface passivation and formation of carrier-selective contacts.^6,11^ However, corrosion over time in aqueous solutions continues to be a major limiting factor of Si photocathodes, especially in the dark under (often untested) open circuit conditions.^12^ Exploration of expanded chemical spaces for new semiconductors may be key to developing the multifunctional layers that are needed for carrier selectivity and protection from degradation.

Ternary nitride materials,^13^ in particular II–IV–N_2_ semiconductors,^14^ have been studied within a limited scope as carrier-selective contacts for crystalline Si PV devices.^15^ For example, ZnSnN_2_ was used as a passivating and/or carrier-selective contact on Si heterojunction solar cells, resulting in an open circuit voltage of 500–550 mV,^16^ and theoretical modeling suggests that further improvements in performance could be realized.^17^ The structural analog ZnSn_1–*x*_Ge_*x*_N_2_, where Sn^4+^ is partially replaced with Ge^4+^, was investigated to provide band edge tunability for Si heterojunction solar cells, but Ge-rich devices produced a lower photovoltage than the ZnSnN_2_ case because low carrier concentrations resulted in charge transfer barriers at the nitride/Si interface.^18^ More recently, it has been shown that p-type Si with a layer of ZnTiN_2_ grown by RF co-sputtering was able to generate a surface photovoltage of >300 mV.^19,20^ However, the photovoltaic response of those devices was weak, possibly because the native SiO_*x*_ layer was not removed from the Si, preventing formation of a good junction. Nevertheless, this recent result indicates that ZnTiN_2_ and Si might form a charge-separating junction, supporting the use of ZnTiN_2_ as an electron-selective contact for Si photocathodes in PEC applications.

We have recently studied wurtzite ZnTiN_2_ for photoelectrode applications due to its ability to form self-limiting surface oxide layers in aqueous solutions.^21^ Specifically, ZnTiN_2_ appears to form Ti- and/or Zn-containing surface oxides depending on pH and electrochemical bias, as expected from its thermodynamic stability in those environments. Ti- and Zn-based oxides are well studied as protective layers on photoelectrodes,^4,22,23^ and their formation *in situ* on the surface of ZnTiN_2_ warrants continued investigation of the durability of this material in aqueous solutions. Despite this promising durable surface chemistry and its suitable, visible range absorption onset (∼2 eV), initial synthetic demonstrations of ZnTiN_2_ have had high n-type carrier concentrations >10^20^ cm^–3^, consistent with degenerate doping.^21,24^ However, high n-type doping is a desirable feature of electron-selective layers, suggesting that ZnTiN_2_ may have dual functionality as a carrier-selective contact and a self-passivating protective layer in PEC applications.

In this work, we deposited ZnTiN_2_ films on p-type Si wafers (ZnTiN_2_/Si) by RF co-sputtering to assess the ability of ZnTiN_2_ to act as an electron-selective and protective layer on Si photocathodes. Regenerative PEC measurements in aqueous methyl viologen electrolytes of different pH and in non-aqueous ferrocene electrolyte revealed a constant open circuit voltage (*V*_OC_) of 400 mV vs the solution potential (*E*_sol_) for ZnTiN_2_/Si, while the *V*_OC_ of bare Si varied from 220 to 480 mV vs *E*_sol_ under the same test conditions. ZnTiN_2_/Si also showed greater durability in the measured *V*_OC_ and fill factor (FF) over 72 h in a 0.1 M KHCO_3_ solution at pH 10.5, compared to bare Si photocathodes. Improved photocurrent and photovoltage stability of ZnTiN_2_/Si was also demonstrated during aqueous methyl viologen reduction at pH 3.5 over 21 h under illumination. Energy-dispersive X-ray spectroscopy (EDS) indicated an increase in oxygen content after PEC operation and a pH-dependent change in the cation ratio, consistent with expectations from the Pourbaix diagram.^21^ The pH-dependent change was also supported by X-ray photoelectron spectroscopy (XPS) data. These results highlight the possibility of using ZnTiN_2_ as a carrier-selective, protective layer on Si photocathodes and offer a broad scope of future research directions for improving understanding and function of these contact layers.

## Results and Discussion

ZnTiN_2_ films in this work were synthesized for PEC testing by previously demonstrated synthetic methods; we refer readers to prior work for further details outside of the brief discussion in the SI.^21,24^ ZnTiN_2_ films were deposited by radiofrequency (RF) co-sputtering onto the polished side of a (100)-oriented p-type Si substrate after removal of the surface oxide with dilute HF. The cation ratio (Zn/(Zn + Ti)) across the film was measured by X-ray fluorescence spectroscopy (XRF). Areas of the film chosen as samples for PEC testing were nominally stoichiometric, having a cation ratio of Zn/(Zn + Ti) = (49±1)%. Structural, morphological, and optoelectronic characterization are shown in Figure S1. Overall, these physical and optoelectronic results are consistent with our previous works on ZnTiN_2_ showing a preferential growth in the (002) direction, a columnar morphology, and an optical bandgap ca. 1.8–2.0 eV.^21,24^ As a result of its high carrier concentration, reference ZnTiN_2_ films grown on n-type GaN under similar conditions behaved like conductors rather than semiconductors in PEC current-voltage sweeps, showing no obvious photocurrent or photovoltage contribution from ZnTiN_2_ (Figure S2).

### Demonstration of ZnTiN_2_–Si Heterojunction

Regenerative PEC measurements were performed to study the photocurrent and photovoltage characteristics of unprotected p-type Si photocathodes (hereafter, Si) and Si photocathodes with a ZnTiN_2_ surface layer (hereafter, ZnTiN_2_/Si). In regenerative photoelectrochemistry, a semiconductor photoelectrode is placed in contact with a solution that contains an outer-sphere, single-electron redox couple to easily create a charge carrier-separating junction without the kinetic complications of multi-electron reactions.^25^ In our data, all working electrode potentials are referenced to *E*_sol_ set by the concentrations of the reduced and oxidized species of the redox couple. The *V*_OC_ for test photoelectrodes is quoted in mV as shorthand for “mV vs *E*_sol_.” The ferrocenium/ferrocene redox couple (Fc^+/0^, simply referred to as “Fc”) was prepared by dissolving the reduced and oxidized forms in acetonitrile (0.5 mM Fc^+^, 10 mM Fc^0^). The methyl viologen couple (MV^2+/+^, simply referred to as “MV”) was prepared *in situ* by pre-electrolysis of MV^2+^ in water to reach approximate concentrations of 49.75 mM and 0.25 mM of MV^2+^ and MV^+^, respectively (calculated from the electrochemical potential of −0.507 V vs Ag/AgCl of the resulting electrolyte).^26^ Under regenerative conditions such as these with a well-defined solution potential, the *V*_OC_ of the illuminated semiconductor electrode is taken to be its photovoltage.

**Figure 1:**
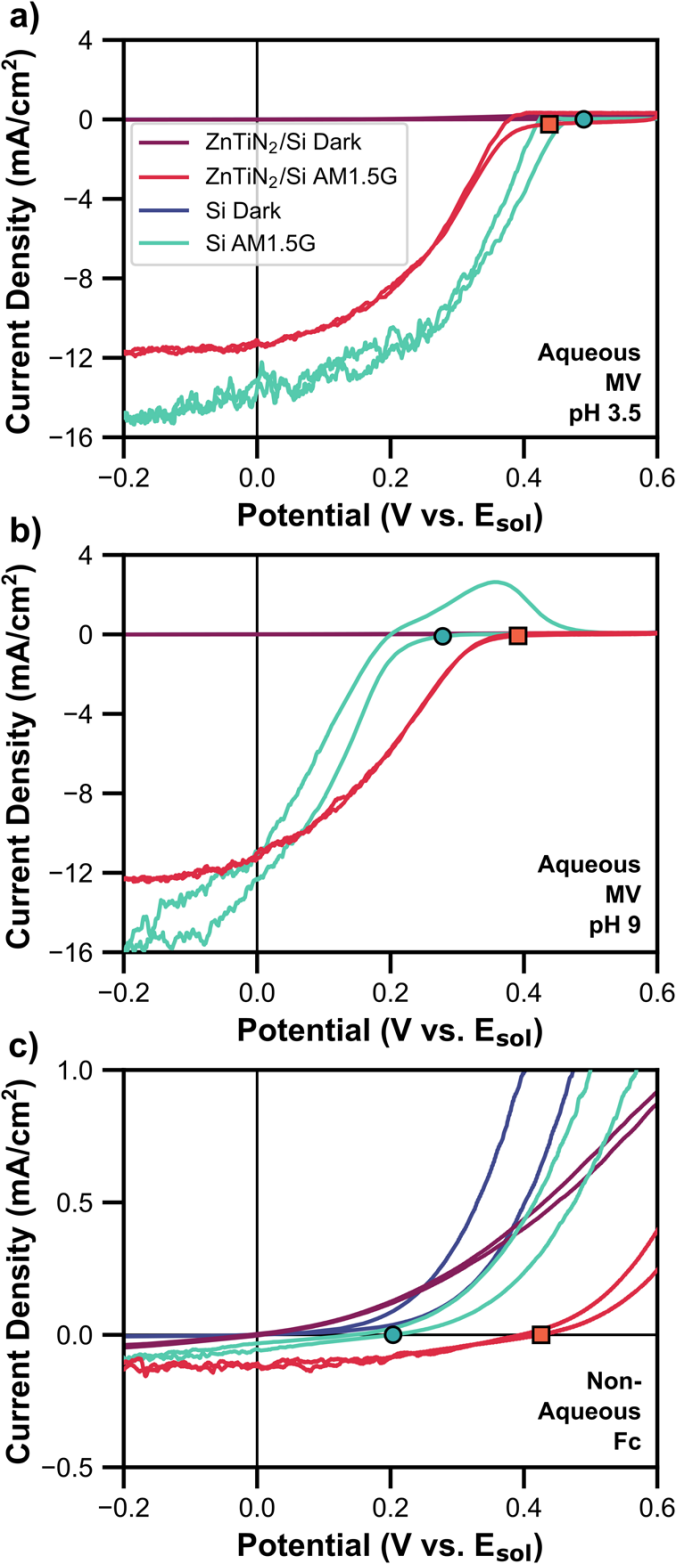
Cyclic voltammograms for ZnTiN_2_/Si (red lines) and bare Si (teal lines) in (a) aqueous methyl viologen (MV) at pH 3.5 and (b) pH 9, and (c) non-aqueous ferrocenium/ferrocene (Fc) solutions in the dark and under simulated AM1.5G illumination. Reference electrode (RE) was a small carbon cloth for aqueous measurements and a Pt wire for non-aqueous measurements. Counter electrode (CE) was a large carbon cloth for aqueous measurements and a Pt mesh for non-aqueous measurements. Scan speed was 50 mV/s, and the forward scan proceeded from +0.6 V to −0.2 V. Square (ZnTiN_2_/Si) and circular (Si) markers indicate the *V*_OC_ of the photoelectrode on the forward scan.

Cyclic voltammetry scans (CVs) of ZnTiN_2_/Si and bare Si in aqueous MV and non-aqueous Fc solution are shown in Figure 1 with markers indicating the *V*_OC_ (a note about determining the *V*_OC_ is found in the SI). Under all conditions, ZnTiN_2_/Si and Si both produced negative (reducing) photocurrent under illumination and their illuminated open circuit voltages moved to more positive potentials compared to the dark. This indicates that they both act as photocathodes. We note that ZnTiN_2_/Si showed a lower photocurrent than Si due to parasitic absorption by ZnTiN_2_, which is expected based on the absorption onset of ZnTiN_2_ (Figure S1d) and is discussed further below. In pH 3.5 MV, the *V*_OC_ for Si and ZnTiN_2_/Si was 490 mV and 440 mV, respectively. In pH 9 MV, the Si *V*_OC_ decreased to 290 mV, while the ZnTiN_2_/Si remained similar at 390 mV for a single measurement (average of multiple measurements shown and discussed below). In non-aqueous Fc, the Si *V*_OC_ further decreased to 200 mV, while ZnTiN_2_/Si remained at 430 mV. The significantly lower photocurrent for both photocathodes in Fc is due to the low concentration of the reducible Fc^+^ at 0.5 mM.

The three electrolyte conditions chosen here help characterize the nature of the carrier-separating junction (solid–liquid vs solid–solid) for each electrode type. For a solid–liquid junction with MV, the *V*_OC_ of Si varies with pH because the energy band positions of Si depend on the pH, while the electrochemical potential of MV is independent of the pH, as no protons or hydroxide ions are involved in the redox reaction.^27^ This leads to a larger difference between the Fermi level of p-type Si and the electrochemical potential of MV at low pH compared to high pH, leading to more band bending and a larger photovoltage at low pH. As expected, this pH dependence was observed for the bare Si photocathode. The *V*_OC_ of ZnTiN_2_/Si did not change with pH, which indicates the presence of a solid-state heterojunction (i.e., the voltage is generated by the ZnTiN_2_/Si interface). In non-aqueous Fc, a small photovoltage from the bare Si photocathode is expected and observed because Fc is known to make a poorly rectifying junction with p-type Si.^28^ This is again a result of the difference between the Fermi level of the photoelectrode and the electrochemical potential of the redox couple. ZnTiN_2_/Si preserved its *V*_OC_ in these conditions, further supporting the interpretation that a solid-state heterojunction has formed.

Figure 2a shows the average *V*_OC_ for ZnTiN_2_/Si and bare Si across the three different redox couple and electrolyte conditions for multiple samples. While the *V*_OC_ of Si varied by 260 mV across the three solutions, the *V*_OC_ of ZnTiN_2_/Si remained steady within error around 400 mV vs *E*_sol_. For a solid–liquid junction, the amount of charge carrier separation (i.e., photovoltage) depends on the solution potential (determined by the redox couple and the pH) and the Fermi level position (e.g., p-type vs n-type doping) of a given semiconductor sample.^29^ Since the *V*_OC_ of ZnTiN_2_/Si is independent of the solution potential, the main carrier separation mechanism cannot be the solid-liquid junction, but rather a solid-state junction, which has similarly been shown for Si photocathodes with a degenerate n^+^-Si layer on the surface.^27^ This demonstrates that ZnTiN_2_ and Si have formed a carrier-separating heterojunction, with the ZnTiN_2_ facilitating electron movement toward the photoelectrode surface, as depicted qualitatively in Figure 2b. Having a *V*_OC_ independent of *E*_sol_ is desirable for photoelectrodes that will perform fuel-forming reactions because the attainable *V*_OC_ would otherwise be limited by the given semiconductor-redox couple pair.^29^

**Figure 2:**
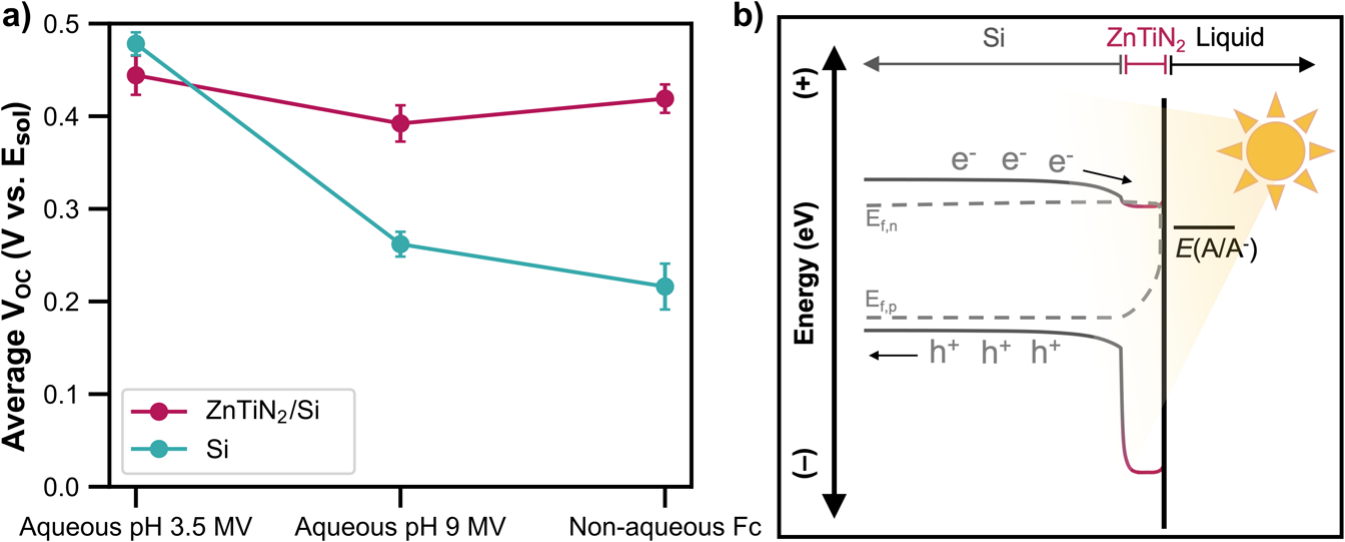
(a) Plot of the open circuit voltage (V vs *E*_sol_) of ZnTiN_2_/Si and Si photoelectrodes in designated redox couple solutions under simulated AM1.5G illumination. Raw data for representative scans can be found in Figure 1 and contents of electrolyte solutions are described in the Methods section. (b) Schematic energy band diagram qualitatively depicting the junction between ZnTiN_2_ and p-type Si in contact with a redox couple under illumination. Minimal band bending is expected in the ZnTiN_2_ due to the high carrier concentration.

Although the ZnTiN_2_ layer has not been optimized as an electron-selective layer, the photovoltage is nonetheless promising. For example, as seen in the absorption spectrum in Figure S1d, ZnTiN_2_ absorbs light strongly above 2 eV. The ZnTiN_2_ films were 180–190 nm thick, as determined by ellipsometry and confirmed by cross-sectional SEM (Figure S4), meaning that at 2.75 eV about 98% of the light is absorbed and cannot reach the Si. This reduces the ZnTiN_2_/Si photocurrent at shorter wavelengths (confirmed by incident photon to current efficiency measurements, Figure S5). This certainly contributes to the lower photocurrent observed for ZnTiN_2_/Si in Figure 1. Transient reflectance measurements confirmed that the carrier lifetime of Si was the same in ZnTiN_2_/Si and Si photoelectrodes (18 μs), so the film deposition process did not evidently degrade the optoelectronic qualities of the photoabsorber (Figure S6). It may be possible to limit optical losses by using a thinner ZnTiN_2_ layer, although the stability and charge transport implications of a thinner layer would then need to be assessed. We also note that in a fuel-forming system, a Si photoabsorber would need to operate in tandem with a wide bandgap photoabsorber to generate sufficient photovoltage to drive the hydrogen evolution reaction or the CO_2_ reduction reaction in concert with oxygen evolution.^30^ The photocurrent obtained in ZnTiN_2_/Si is thus relevant to more realistic PEC systems, unlike the photocurrent from Si alone.

### Stability of ZnTiN_2_/Si Photoelectrodes

The ability of ZnTiN_2_ to act as a protective coating as well as an electron-selective contact layer for Si was assessed through comparative PEC durability studies with unprotected Si. Photoelectrode performance was assessed by periodically taking CVs over a 72-hour period in a regenerative pH 9 MV PEC cell. In between CVs, the photoelectrodes were “stressed” by placing them in a 0.1 M KHCO_3_ electrolyte at pH 10.5 without N_2_ purging, and without electrical connection to the potentiostat (an ungrounded condition). This “stress” solution was chosen to have a bicarbonate concentration and pH relevant to CO_2_ reduction reaction (CO_2_RR) conditions during operation, where the H^+^ concentration can significantly decrease at the electrode surface.^31,32^ The CVs taken at each time point for each electrode are shown in Figure 3a (only cathodic scan shown for clarity). The onset of the photocurrent for Si is shown to shift to more cathodic potentials over time, degrading the *V*_OC_ and FF. This is due to the formation of highly resistive surface SiO_*x*_ between the photoelectrode and the solution, requiring higher applied bias to extract charge carriers.^33^ By contrast, the shape of the ZnTiN_2_/Si current-voltage curve is more stable, with minimal change to the *V*_OC_ and FF. This demonstrates protection to the underlying Si. The quantitative change in the *V*_OC_ for ZnTiN_2_/Si and Si over 72 h is shown in Figure 3b. The *V*_OC_ of ZnTiN_2_/Si only slightly decreases, by about 10 mV over 72 h, with a relatively flat continuing trend. By contrast, the Si *V*_OC_ shows a downward trend going from ca. 240 mV to ca. 200 mV after 72 h. These results demonstrate a protective function of ZnTiN_2_ in addition to the added charge carrier separation provided by the layer.

**Figure 3:**
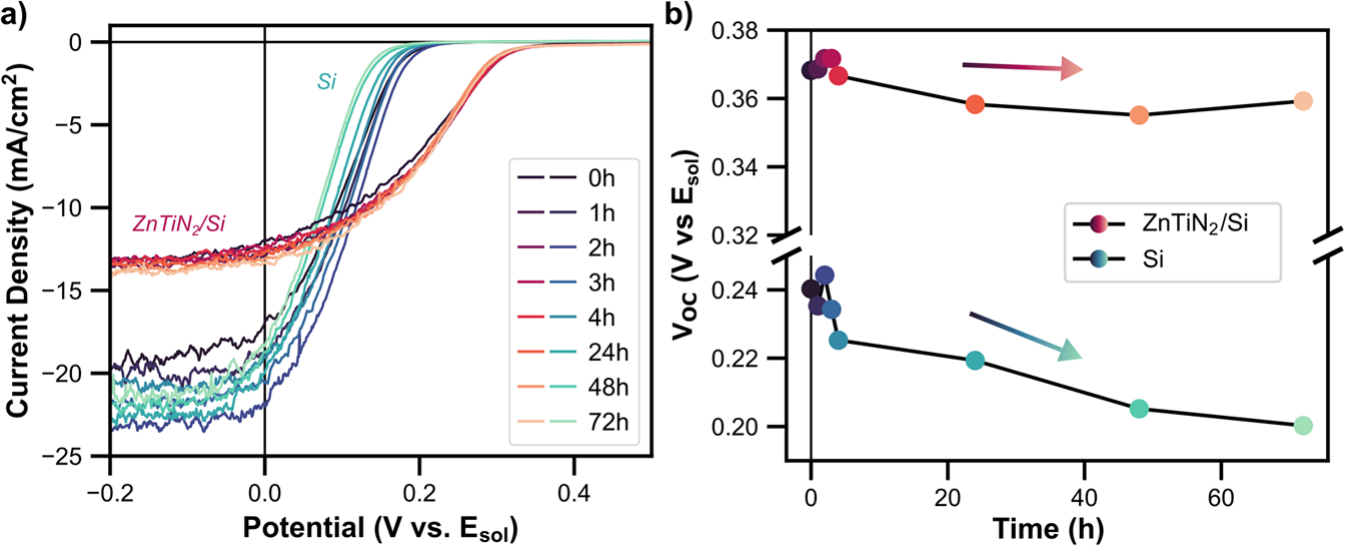
Dark open circuit stability studies: (a) Current-voltage curves of ZnTiN_2_/Si and Si in pH 9 aqueous MV electrolyte under AM1.5G illumination. Electrodes were stored in 0.1 M KHCO_3_ solution at pH 10.5 without N_2_ purging for the designated amounts of time in between measurements in the MV solution. Electrodes were disconnected from the potentiostat while stored in 0.1 M KHCO_3_. (b) Open circuit potential (*V*_OC_) vs *E*_sol_ of ZnTiN_2_/Si and Si in pH 9 MV solution after storage in pH 10.5, 0.1 M KHCO_3_ solution for the designated amounts of time. The colors correspond to the same time points across the two panels.

The stability of ZnTiN_2_/Si and Si was further compared under active MV reduction conditions. Chronoamperometry was performed at +0.1 V vs *E*_sol_ under AM1.5G illumination for 21 h in pH 3.5 MV solution; the normalized photocurrent over time is shown in Figure 4a. The current for ZnTiN_2_/Si decreased by 7% and Si by 19%. CVs were taken at designated time points and forward cathodic scans are plotted in Figure 4b. Si showed a notable decrease in *V*_OC_ and FF within the first hour, and then a second large decrease occurred between hour 6 and 21. By contrast, ZnTiN_2_/Si showed a minor decrease in photocurrent in the first hour but remained relatively unchanged over the course of the 21 h. The quantitative change in *V*_OC_ over 21 h is shown in Figure 4c. The *V*_OC_ for ZnTiN_2_/Si and Si decreased by about 12 mV and 120 mV, respectively. These data further support a protective effect of the ZnTiN_2_ and demonstrate the stability of the layer. Further testing under fuel-forming conditions with a catalyst will be necessary to see how the protective effect extends to full PEC device operation. However, for this initial indication of how well ZnTiN_2_ acts as a barrier on Si, we sought to avoid the possible contributions of catalyst fouling or delamination to device instability.

**Figure 4:**
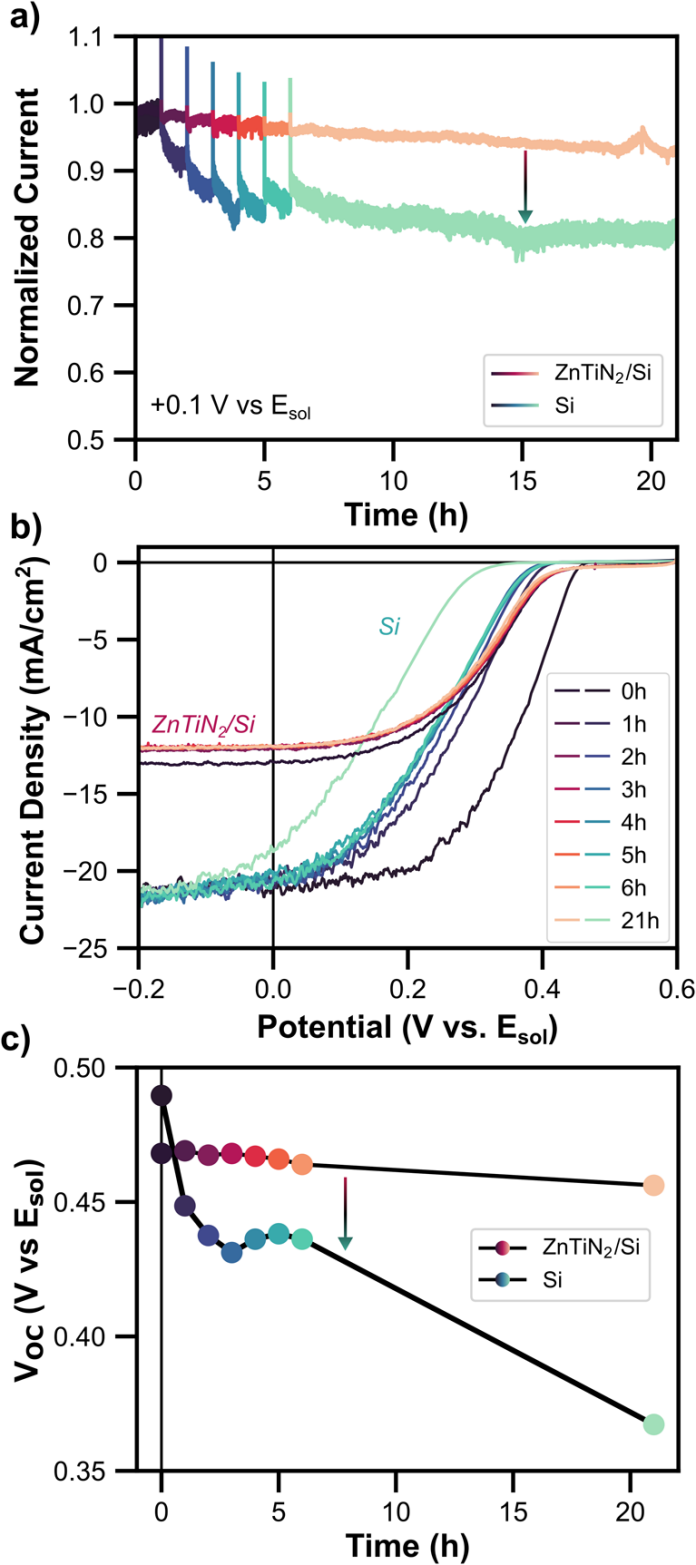
Illuminated chronoamperometry durability studies: (a) Normalized photocurrent for ZnTiN_2_/Si and Si under simulated AM1.5G at +0.1 V vs *E*_sol_ in pH 3.5 aqueous MV. (b) Current-voltage curves at designated time points during the illuminated chronoamperometry experiment. (c) Plot of the *V*_OC_ over the course of the chronoamperometry experiment. The colors correspond to the same time points across the three panels.

### Elemental Analysis

Previous work characterizing ZnTiN_2_ surfaces by X-ray photoelectron spectroscopy (XPS) before and after exposure to electrolyte indicated that ZnTiN_2_ forms protective surface oxides in aqueous solutions under pH and applied bias conditions that are relevant for fuel-forming reactions.^21^ To further investigate the formation of surface layers in response to the photoelectrochemical conditions used here, XPS and EDS were performed on ZnTiN_2_/Si samples to determine respective surface and bulk elemental compositions after cumulative exposure to electrolyte for 1 h at open circuit in the dark and 1 h at 0 V vs *E*_sol_ under illumination in pH 3.5 or pH 9 MV. Figure 5a qualitatively depicts the estimated probe depth of XPS and EDS in relation to the sample thickness. In these samples, a tape mask was used to define the area exposed to electrolyte, rather than epoxy (modified electrode fabrication scheme in Figure S7). Surface-sensitive XPS used a ZnTiN_2_/Si film that was not made into an electrode or exposed to electrolyte as a control due to probable surface contamination by the tape adhesive. For more bulk-sensitive EDS, an area covered by tape and never exposed to electrolyte was compared to an area exposed to electrolyte on the same electrode.

**Figure 5:**
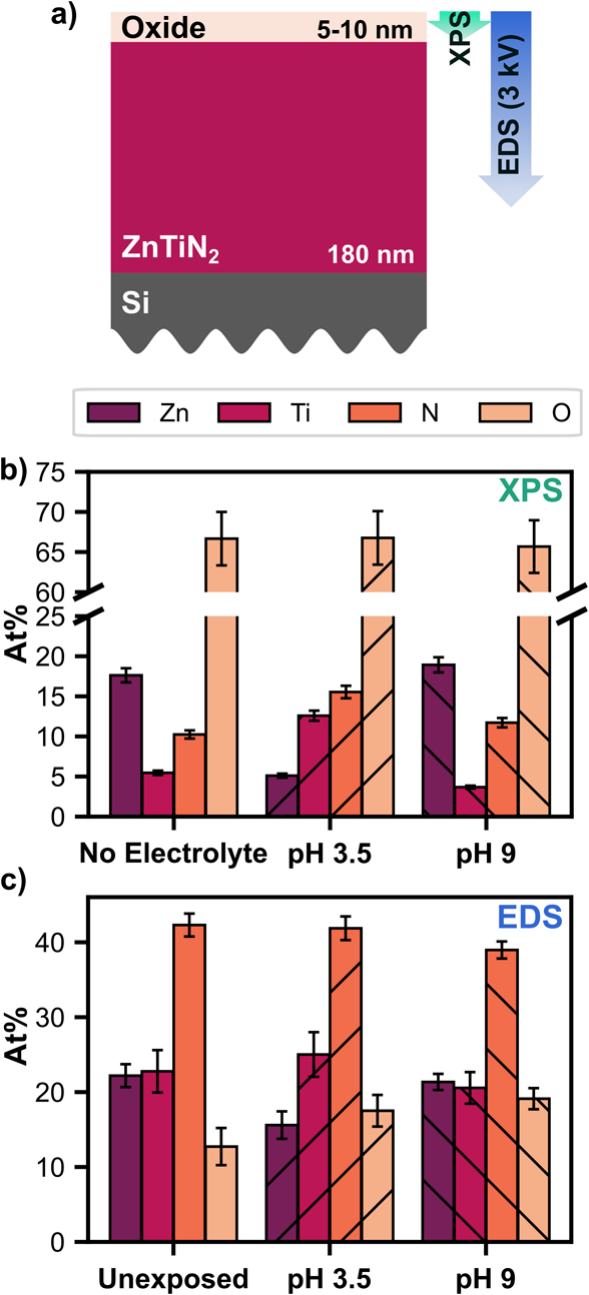
(a) Schematic diagram depicting the material layers in ZnTiN_2_/Si with approximate thickness and approximate probe depth of XPS and EDS measurements. (b) Bar plot depicting the surface elemental composition of Zn, Ti, N, and O by XPS on a ZnTiN_2_/Si film that was not made into an electrode or placed in electrolyte (“No electrolyte”; left) and on ZnTiN_2_/Si photoelectrodes after PEC operation in pH 3.5 (middle) and pH 9 (right) MV electrolyte. (c) Bar plot depicting the bulk elemental abundance of Zn, Ti, N, and O by EDS on ZnTiN_2_/Si photoelectrodes without exposure to electrolyte (“Unexposed”; left) and after photoelectrochemical operation in pH 3.5 (middle) and pH 9 (right) MV electrolyte. Data were taken using an accelerating voltage of 3 kV. All data were normalized to the sum of Zn, Ti, N, and O.

Surface elemental composition data from XPS are shown in Figure 5b for Zn, Ti, N, and O, normalized to the sum of these elements (full results provided in Table S1). The no electrolyte control sample was Zn-rich, consistent with our previous work showing a Zn-rich surface on as-grown ZnTiN_2_.^21^ After exposure to pH 3.5 MV, Zn became less prevalent on the surface, and after PEC operation in pH 9 MV, there was minimal change in the surface cation concentrations. The oxygen content was high in all samples. High surface oxygen content on the no electrolyte sample is likely due to handling and storage in air for long periods of time (days). Beyond elemental composition, high resolution XPS spectra revealed slight changes to the cation and anion bonding environments at the film surface depending on electrolyte pH **(** Figure S8). A brief discussion of specific pH-dependent changes is included in the SI. Post-PEC grazing incidence XRD also demonstrated significant pH-dependent changes in crystalline phases present on the surface, although we leave identification of specific Zn–Ti–O–N phases to future work (Figure S9).

EDS was used to probe further into the sample bulk, and the elemental abundance of Zn, Ti, N, and O (normalized to the sum of those elements) of ZnTiN_2_/Si exposed to pH 3.5 and pH 9 electrolyte are shown in Figure 5c. EDS results from an area unexposed to electrolyte are presented for comparison. Consistent with the XPS results, the area tested in pH 3.5 MV showed a reduction in the amount of Zn compared to the unexposed area, while the sample tested in pH 9 MV showed no significant change in the cation composition compared to the unexposed area. Both areas exposed to electrolyte showed a higher O content. EDS was performed at accelerating voltages of 3 kV and 10 kV for increased surface sensitivity and improved quantitative certainty, respectively. The same trends in elemental composition were observed for both accelerating voltages, and values obtained at 10 kV are provided in the SI (Figure S10; full elemental percentages in Table S2).

The elemental composition of the films without electrolyte exposure or PEC operation were consistent with previous findings for ZnTiN_2_.^21^ XPS data revealed a Zn-rich surface, while EDS data showed the expected cation ratio (Zn/(Zn + Ti)) for ZnTiN_2_. XPS showed a high oxygen concentration regardless of electrolyte exposure, as surface oxidation upon exposure to atmospheric oxygen is well documented for nitride films, and for ZnTiN_2_ in particular.^21,34,35^ Surface oxidation in nitrides can be associated with a passivating effect that limits further film oxidation,^35^ and this expected passivating effect underlies our interest in this nitride for photoelectrochemical applications.^21^ Oxygen was also present in the EDS measurements, although the exact location of the oxygen cannot be directly determined using this method. The oxygen content measured by XPS was higher than that measured by EDS, suggesting that oxygen is distributed more toward the surface, but incorporation of oxygen into the bulk cannot be ruled out. Prior works on RF sputtered TiN and Zn_3_N_2_—as well as other ternary nitrides—show that bulk oxygen incorporation is a common feature of this film growth technique.^24,36–38^ The presence of some bulk oxygen in our films is expected to influence the n-type carrier concentration of the ZnTiN_2_, although the precise relationship between oxygen incorporation and carrier concentration in nitride films is still a matter of debate and may be material-dependent.^24,38–40^ Nonetheless, oxygen incorporation may affect the electron-selective function of the ZnTiN_2_.

After PEC operation, the XPS and EDS results revealed changes in the elemental composition and bonding that are consistent with the predicted Pourbaix diagram, in line with our previous work on electrochemical polarization of ZnTiN_2_ films.^21^ This was reflected in the change of the cation ratio measured by EDS from 0.49 in the unexposed area to 0.38 after exposure to pH 3.5, as anticipated for a more TiO_2_-like surface. The cation ratio remained relatively unchanged after exposure to pH 9 (cation ratio of 0.49 unexposed vs 0.51 exposed), consistent with a mixed Ti_3_Zn_2_O_8_-like and ZnO-like surface. The cation ratio from XPS measurements showed a similar trend of Ti enrichment after operation in pH 3.5 (cation ratio 0.29), and showed Zn enrichment after operation at pH 9 (cation ratio 0.84), consistent with previous work.^21^ High resolution XPS spectra of Ti 2p and O 1s (Figure S8b and S8d) provide further support for the formation of TiO_2_ in particular at the surface after operation at pH 3.5. Compared to EDS data, XPS data showed a more pronounced deviation from the expected cation ratio of 0.5 for ZnTiN_2_, indicating that chemical changes in response to PEC operation occur primarily at the surface. The higher oxygen content measured by XPS compared to EDS also suggests that there is more oxide distributed toward the surface of the ZnTiN_2_. However, EDS shows an increase in oxygen content after PEC operation, while this is not observed in XPS, indicating that the oxide layer that formed under atmospheric ambient is at least comparable to the probe depth of XPS and grows slightly thicker after PEC operation (in addition to the compositional changes discussed). Paired with the durability data, the results suggest that the insoluble oxides predicted to form according to the Pourbaix diagram lend a protective effect to the photoelectrodes.

These data indicate that surface oxides form on ZnTiN_2_, but as shown in the PEC data, they do not appear detrimental to charge transfer, unlike what would be expected for highly insulating SiO_*x*_.^41^ Of course, *in operando* measurements would be required to achieve a more detailed understanding of oxide formation under reaction conditions, but the intense method development required to reliably perform such experiments is beyond the scope of this preliminary study. While our durability data support the formation of protective surface oxides, the small cathodic current seen before the onset of MV reduction in the CVs also suggests that slow reactions may be taking place that could be causing potential-dependent changes in the ZnTiN_2_ film. Our EDS data possibly indicate some thinning of the film in areas exposed to electrolyte compared to areas not exposed to electrolyte, as the relative Si signal appears higher in the electrolyte-exposed areas (see discussion in SI, Table S3). However, atomic force microscopy (AFM) does not show a significant change in roughness for ZnTiN_2_/Si exposed to electrolyte compared to an as-grown ZnTiN_2_/Si film (Figure S11), and overall, the films remain rather smooth, with post-PEC roughness less than 10 nm. Future studies will benefit from investigating the evolution of surface oxides and film material properties over PEC tests of longer duration and under fuel-forming conditions.

There are a number of possible avenues for further studying and tuning the properties of ZnTiN_2_ as a selective contact and protective layer for Si photocathodes. The silicon used here was test grade,^42^ while higher quality, lower defect density silicon is used for PV devices. Studies with higher quality Si may better elucidate the solid-state junction properties of the ZnTiN_2_/Si. Additionally, GaIn eutectic was used to create the ohmic back contact for all photoelectrodes, while more hole-selective layers would be expected to further improve the photovoltage.^43,44^ The development and synthesis of ZnTiN_2_ is also in early stages with many possible routes for further study and improvement. Previous work on ZnTiN_2_ shows that careful selection of film synthesis conditions may tune the carrier concentration of films.^24^ Improved synthetic control over bulk oxygen content could help clarify the relationship between oxygen content and carrier density. Adjusting the carrier concentration could have implications for the electron-selectivity of films, surface reaction kinetics (and thus photocurrent), or the sub-bandgap absorption and resultant parasitic absorption. High carrier concentrations in ZnTiN_2_ were a motivator for studying the material as an electron-selective layer; however, the visible-range bandgap of ZnTiN_2_ likewise makes it an interesting photoabsorber. Once carrier concentrations in ZnTiN_2_ can be well controlled and optimized, this could open the possibility of a ZnTiN_2_/Si tandem photoelectrode, as the materials can clearly be electrically integrated. Further studies under fuel-forming conditions will also be essential for a realistic understanding of this material in a PEC device.

## Conclusion

In this work, ZnTiN_2_ was investigated as a protective, electron-selective layer on p-type Si photocathodes using regenerative photoelectrochemistry. ZnTiN_2_/Si demonstrated a constant open circuit voltage of 400 mV vs the solution potential across different electrolyte conditions, indicating the formation of a carrier-separating junction at the interface between ZnTiN_2_ and Si. Upon prolonged exposure to 0.1 M KHCO_3_ at pH 10.5 in the dark, ZnTiN_2_/Si showed a stable *V*_OC_ and fill factor, in contrast to bare Si which degraded under the same conditions. ZnTiN_2_/Si also showed greater durability under illumination while performing methyl viologen reduction in aqueous solution. Post-PEC characterization revealed the formation of surface oxides that enable the durability found in this study. While the ZnTiN_2_ parasitically absorbs light, shading the underlying Si, this could be improved by thinning the ZnTiN_2_ and gives an indication of how the Si would function in a tandem configuration. Future studies should evaluate the implications of material quality (including defect concentration and cation ratio) on junction performance. Overall, this work offers an exciting new direction for studying the optoelectronic functionality of ternary nitride materials.

## Methods

### Materials

Methyl viologen dichloride hydrate (MV^2+^; 98%, Aldrich or Thermoscientific), potassium sulfate (≥99.0%, Sigma-Aldrich), potassium phosphate monobasic (≥99.0%, Sigma-Aldrich), potassium phosphate dibasic (99.95%, Sigma-Aldrich), potassium hydrogen phthalate (KHP; 99.95%, Sigma-Aldrich), potassium carbonate (99.7%, Sigma-Aldrich), potassium hydroxide (Certified ACS, Fisher Scientific), hydrochloric acid (36.5-38%, J. T. Baker), and tetrabutylammonium hexafluorophosphate (TBAPF_6_; > 98%, TCI) were used as-received. Ferrocene (Fc^0^; 98%, Millipore Sigma) was purified by sublimation. Ferrocenium tetrafluoroborate (Fc^+^; technical grade, Millipore Sigma) was purified by recrystallization by dissolving in acetone and using diethyl ether as an antisolvent. Acetonitrile (≥99.9%, Sigma-Aldrich) was distilled before use. Reagents used in non-aqueous photoelectrochemistry (i.e., acetonitrile, TBAPF_6_, Fc^0^, and Fc^+^) were stored in a glovebox after purification (if any was performed). Water was purified to 18.2 MΩ-cm resistivity for aqueous photoelectrochemical solutions.

Single-side polished, (001)-oriented, test-grade p-type silicon wafers doped with boron were purchased from University Wafers with 620 μm thickness and resistivity of 0–10 Ω-cm quoted by the manufacturer. This resistivity range corresponds to a dopant density range of approximately 1 × 10^15^ to 1 × 10^17^ cm^–3^.

### ZnTiN_2_ Synthesis

Prior to film deposition, native surface oxide was removed from the Si substrate by dipping in a dilute (<10%) aqueous HF solution. ZnTiN_2_ films were deposited onto the polished side of a stationary 50 x 50 mm p-type Si substrate by radio-frequency (RF) co-sputtering in a custom vacuum chamber. A mask was placed over the substrate such that there were 4 separate rows of ZnTiN_2_ deposited on the substrate. The row height was 11 mm. A chamber base pressure of <1 × 10^–7^ Torr was reached before 20 sccm Ar and 10 sccm N_2_ were added to the chamber, after which process chamber pressure was maintained at 4 × 10^–3^ Torr. 120 W was applied to the Ti target and 150 W was applied to the ZnTi target. No intentional heating was applied during the deposition.

### Physical Characterization

X-ray diffraction (XRD) patterns were taken using a Bruker D8 Discover (Cu Kα source) with an area detector over a range of 2θ = 19–77° and χ = 60–120°. Data was integrated over χ to plot intensity vs 2θ. X-ray fluorescence spectroscopy (XRF) was used to measure the ratio of Zn:Ti in the film and samples were taken from film areas that were nominally 50:50 (±2%) Zn:Ti. The tilted scanning electron micrograph shown in Figure S1c was taken in a Solaris FIB-SEM (TESCAN) at 2 kV accelerating voltage and 100 pA beam current. Scanning electron microscopy with EDS was performed in a Nova NanoSEM 630 (FEI) using an Oxford Instruments Ultim Max detector and AZtec software. The cross-section image in Figure S4 was taken at 3 kV beam voltage and 1.3 nA beam current. EDS on samples for pre- and post-PEC characterization were taken at accelerating voltages of 3 kV (1.3 nA beam current) or 10 kV (2.6 nA beam current), as denoted in the text. XPS data were obtained on a PHI VersaProbe III using Al Ka radiation (1486.7 eV). The XPS data were calibrated with Au and/or Cu metal, which was cleaned via Ar-ion sputtering. The raw atomic concentration has a 5% error due to surface inhomogeneities, surface roughness, literature sensitivity values for peak integration, *etc*.

Ellipsometry was performed on a J. A. Woollam Co. M-2000 variable angle ellipsometer. Raw Ψ and Δ information was collected at 65°, 70°, and 75° over a wavelength range of 0.73–6.46 eV. Data was modeled and fit in the CompleteEASE software package provided by the manufacturer (version 6.69). A generalized oscillator model was constructed, which contained a PSemi-M0 oscillator to model the ZnTiN_2_ optical absorption edge and a Drude oscillator to model the sub-bandgap free carrier absorption. The percentage of light absorbed by ZnTiN_2_ at a particular wavelength was estimated using the Bouguer-Beer-Lambert equation^45^ using the absorption coefficient (*α*) determined by spectroscopic ellipsometry:

$$\frac{I\left(d\right)}{I_{0}}=e^{-\alpha d}$$

where *d* is the thickness of the ZnTiN_2_ film, *I*(*d*) is the illumination intensity at the back of the ZnTiN_2_ film, and *I*_0_ is the illumination intensity at the front of the ZnTiN_2_ film.

Transient reflectance measurements were completed in a pump-probe configuration using a Ti:sapphire regenerative amplifier (Coherent Astrella) with an 800 nm fundamental, a 1 kHz repetition rate and 90 fs pulse width. The pump (1.6 eV) was generated in an optical parametric amplifier (Light Conversion, TOPAS-C). The probe pulse is created through continuum generation in a diode-laser pumped photonic crystal fiber (EOS, Ultrafast Systems). The probe was reflected off the surface of the sample at a 45° angle and directed to the detector, which is a fiber-coupled multi-channel spectrometer with a CMOS sensor. The pump and probe were focused and spatially overlapped at the sample. The pump-probe delay is controlled electronically and data were collected using software provided by Ultrafast Systems. Data were analyzed using Surface Xplorer (Ultrafast Systems).

Surface topography and roughness measurements were collected by PeakForce Tapping atomic force microscopy (AFM) using a Dimension Icon AFM (Bruker Corp.) in ScanAsyst mode. All measurements were conducted with a ScanAsyst-Air Probe (nominally *k* = 0.4 N/m, *f*_*0*_ = 70 kHz, *r*_tip_ = 2 nm, Bruker Corp.) and a peak force set point of 5.000 nN. Nanoscope Analysis software (Bruker Corp.) was used to remove sample tilt from the images by first order image flattening. The root mean square average roughness, *R*_q_, was then calculated for each image using the same software.

### Standard Down-Facing Photoelectrode Fabrication

5 x 5 mm pieces of ZnTiN_2_/Si were cut from sample rows that were determined to have a near-stoichiometric cation ratio (ca. 50:50 Zn:Ti). ZnTiN_2_ film coverage was continuous across the surface of the ZnTiN_2_/Si photoelectrodes. 5 x 5 mm pieces of the uncoated Si substrate were also prepared as comparison electrodes following the same procedure. Gallium–indium eutectic was scribed into the unpolished side of the Si substrate to form an ohmic contact. Silver paste was used to attach a coiled wire, which was insulated by threading through a glass tube. The electrode back and edges were sealed with Loctite EA 9460 epoxy, leaving an active geometric area of approximately 0.05–0.1 cm^2^. The exact geometric area was measured using ImageJ. The epoxy was allowed to cure for at least 2 days before electrodes were photoelectrochemically tested.

### Modified Down-Facing Deconstructable Photoelectrode Fabrication for Post-PEC Characterization

A schematic diagram of the fabrication process is provided in Figure S7. A 4 mm-diameter hole was cut into electroplating tape (3M, 470 Electroplating Tape) with a hole punch, and this was used to mask a 5 x 5 mm piece of ZnTiN_2_/Si. The unpolished side of the Si was scribed with GaIn eutectic. A wire coiled into a flat circle was attached with silver paste, and the edges and back of the Si were epoxied such that only the wire circle was exposed out of the epoxy. At this step, it was essential to make the wire coil completely flat and to ensure the epoxy did not stand taller than the wire circle to aid sample positioning for post-PEC characterization. To perform PEC measurements, a second, longer wire was coiled into a flat circle of similar size as the first, with the rest of the wire being threaded through a glass tube. The two wire coil circles were taped together with electroplating tape such that there was good electrical contact between the sample and the long wire. The tape also covered the back of the Si substrate. To protect the back and sides of the electrode from solution, parafilm was wrapped numerous times around the sample and at the meeting point with the glass tube. The 4 mm-diameter tape mask defined the geometric area of the electrode as approximately 0.13 cm^2^. After photoelectrochemical experiments, the parafilm, tape, and glass tube could be removed to return the sample to a flat geometry (i.e., the semiconductor piece with a flat wire coil epoxied to the back side) for physical characterization.

### Photoelectrochemical Measurements

Si control electrodes were dipped in 10% aqueous HF solution for 60 seconds to remove surface oxide immediately before being placed in solution for testing. ZnTiN_2_/Si electrodes did not receive any initial cleaning or etching steps. Electrodes were placed in a 4-port glass electrochemical cell with a quartz window on the bottom. The vessel was continuously purged with nitrogen throughout experiments. An LED-based solar simulator (Pico, G2V Optics Inc.) was used as the illumination source and was set to the AM1.5G spectrum. An in-house calibrated Si photodiode (FDS100, Thorlabs) was used to measure the illumination power at the sample position in the vessel. The photodiode measured 86% of the photocurrent expected for AM1.5G illumination in the dry PEC vessel.

Three different electrolyte solutions were used for regenerative photoelectrochemistry, designated as aqueous methyl viologen (MV) pH 3.5, aqueous MV pH 9, and non-aqueous ferrocenium/ferrocene (Fc). Photoelectrochemical test solutions consisted of the following: aqueous MV solution at pH 3.5 consisted of 0.5 M K_2_SO_4_ electrolyte, 0.1 M KHP buffer, and 0.05 M MV^2+^ (pH adjusted with concentrated HCl). Aqueous MV solution at pH 9 consisted of 0.5 M K_2_SO_4_ electrolyte, 0.1 M potassium phosphate buffer, and 0.05 M MV^2+^ (pH adjusted with 1 M KOH). Non-aqueous Fc solution contained 0.5 M TBAPF_6_ electrolyte, 0.01 M Fc^0^, and 0.5 mM Fc^+^ in dry acetonitrile (prepared in a glovebox <1 ppm of O_2_ and H_2_O). For obtaining a trend in the *V*_OC_ across electrolytes, more than one electrode sample was measured under each condition.

Aqueous MV experiments required a pre-electrolysis step prior to sample measurements to generate the reduced form of MV^2+^. During pre-electrolysis, a Pt wire inside a fritted glass tube was connected as the counter electrode, a large carbon cloth as the working electrode, and a saturated Ag/AgCl electrode as the reference. A bias of −0.54 V vs Ag/AgCl was applied until a solution potential of approximately −0.51 V vs Ag/AgCl was reached (measured between the Ag/AgCl electrode and a small carbon cloth), allowing for sufficient light to reach the sample while maintaining a well-defined solution potential.

During aqueous sample measurements, a small carbon cloth was used as the reference electrode (poised at the solution potential, *E*_sol_) while a large carbon cloth was used as the counter electrode. For non-aqueous Fc measurements, a Pt wire poised at the solution potential was used as the reference electrode and a Pt wire mesh was used as the counter electrode. The scan speed was 50 mV/s for all CVs, and CVs proceeded from +0.6 V to −0.2 V vs *E*_sol_ on the forward scan. The average *V*_OC_ was determined from at least 3 measurements of the *V*_OC_ from two different photoelectrodes.

### Determining the *V*_OC_ from Current-Voltage Curves of Si and ZnTiN_2_/Si Samples

The open circuit voltage (*V*_OC_) is typically taken as the onset of the photocurrent. For the Fc data, this was determined as the point where the photocurrent crosses the *x*-axis. In the MV solutions, the oxidative (positive) current was nearly zero at applied potentials positive of the oxidation potential of MV^+^, such that searching for the point where current equals zero does not necessarily yield an accurate result. Additionally, ZnTiN_2_/Si displayed a very small (< −0.25 mA/cm^2^) cathodic current before the onset of MV reduction, so the point where the current crosses the *x*-axis in the CV does not correspond to the *V*_OC_. This was confirmed by measuring the potential at open circuit under illumination (Figure S3). Measurements at open circuit conditions were not taken every hour for bare Si photoelectrodes, so the point where the CV inflected downward was chosen as the *V*_OC_. To determine this point, a numerical approximation of the derivative of the current-voltage curve was taken for both samples and a Savitzky-Golay filter was applied to reduce the noise that is inherently introduced by this mathematical procedure. The voltage at which the derivative or slope was equal to 0.1 mAcm^–2^/V was found to reasonably approximate (within 30 mV) and follow the same trend of the *V*_OC_ as measured by other methods (i.e., by the open circuit measurements for ZnTiN_2_/Si and where the CV crossed 0 mA/cm^2^ for Si). Therefore, the *V*_OC_ in MV plotted in Figures 1–4 was taken as the point where the current-voltage curve inflected downward in the cathodic sweep (slope equal to 0.1 mAcm^–2^/V).

### Photoelectrochemical Stability Measurements

For dark electrode stability studies, electrode function was tested by taking a cyclic voltammogram (CV) under simulated AM1.5G illumination at time points designated in the text (0 h being before electrodes were placed in the “stress solution”). The “test solution” in which CVs were performed was pH 9 aqueous MV solution. MV solution was pre-electrolyzed as previously described; the solution potential was −0.510 ± 0.003 V vs Ag/AgCl for all test CVs. In between CVs, electrodes were placed in 0.1 M KHCO_3_ at pH 10.5 (the “stress solution”; pH adjusted with 1 M KOH) in the dark with no electrical connections and no N_2_ purging. For illuminated electrode stability studies, electrodes were placed in pH 3.5 aqueous MV solution as the electrolyte (with pre-electrolysis performed as described above). Chronoamperometry was performed at +0.1 V vs *E*_sol_ under simulated AM1.5G illumination over 21 h. *E*_sol_ remained within 20 mV of the starting value during chronoamperometry. Chronoamperometry was periodically interrupted at the time points designated in the text to perform cyclic voltammetry to assess changes to the power conversion ability of the electrodes. Pre-electrolysis was performed as needed to bring *E*_sol_ back to −0.510 ± 0.003 V vs Ag/AgCl before CVs were taken.

## Supplementary Material



## Data Availability

The raw data underlying this investigation will be made available upon request.
