# Supplementary material for: ZnTiN2 as an Electron-Selective, Protective Layer on Si Photocathodes
Source: ACS Electrochem. 2025 Apr 22;1(6):842–52. doi: 10.1021/acselectrochem.4c00155 (PMC12147160; doi:10.1021/acselectrochem.4c00155)
Supplement: Supplementary file 1 [file ec4c00155_si_001.pdf]

Supporting Information for: **$ZnTiN_2$ as an electron-selective, protective layer on Si photocathodes**

Authors: Anna C. Kundmann,[1,2] John S. Mangum,[2] Mellie Lemon,[2] Maria Kelly,[2,3,4] Dennice M. Roberts,[2] Melissa K. Gish,[2] Elisa M. Miller,[2] Emily L. Warren,[2] Frank E. Osterloh,[1] and Ann L. Greenaway[2]

[1]Department of Chemistry, University of California, Davis, California 95616, United States

[2]Materials Chemical and Computational Science Directorate, National Renewable Energy Laboratory, Golden, Colorado 80401, United States

[3]Department of Chemical and Biological Engineering, University of Colorado Boulder, Boulder, Colorado 80309, USA

[4]Renewable and Sustainable Energy Institute, University of Colorado Boulder, Boulder, Colorado 80309, USA

***Corresponding Authors:** Ann L. Greenaway (ann.greenaway@nrel.gov), Anna C. Kundmann (ackundmann@ucdavis.edu)*

Table of Contents

Physical and Optoelectronic Characterization of $ZnTiN_2$ film on Si 2

Current-Voltage Behavior of $ZnTiN_2$ Grown on n-GaN 3

Determining the V_{OC} from Current-Voltage Curves of Si and $ZnTiN_2$/Si Samples 4

Cross-sectional SEM to Corroborate Film Thickness 5

Incident Photon-to-Current Efficiency (IPCE) Measurement 6

Transient Reflectance (TR) Measurement 7

"Deconstructable" Photoelectrode Preparation 7

XPS Characterization of $ZnTiN_2$/Si 8

GIXRD of $ZnTiN_2$ Photoelectrodes after PEC Operation at pH 3.5 and pH 9 11

Energy Dispersive X-Ray Spectroscopy (EDS) of deconstructable electrodes 11

Note on EDS quantification of Si on $ZnTiN_2$/Si electrodes in unexposed vs exposed areas 12

Atomic force microscopy (AFM) roughness of $ZnTiN_2$/Si after exposure to electrolyte 13

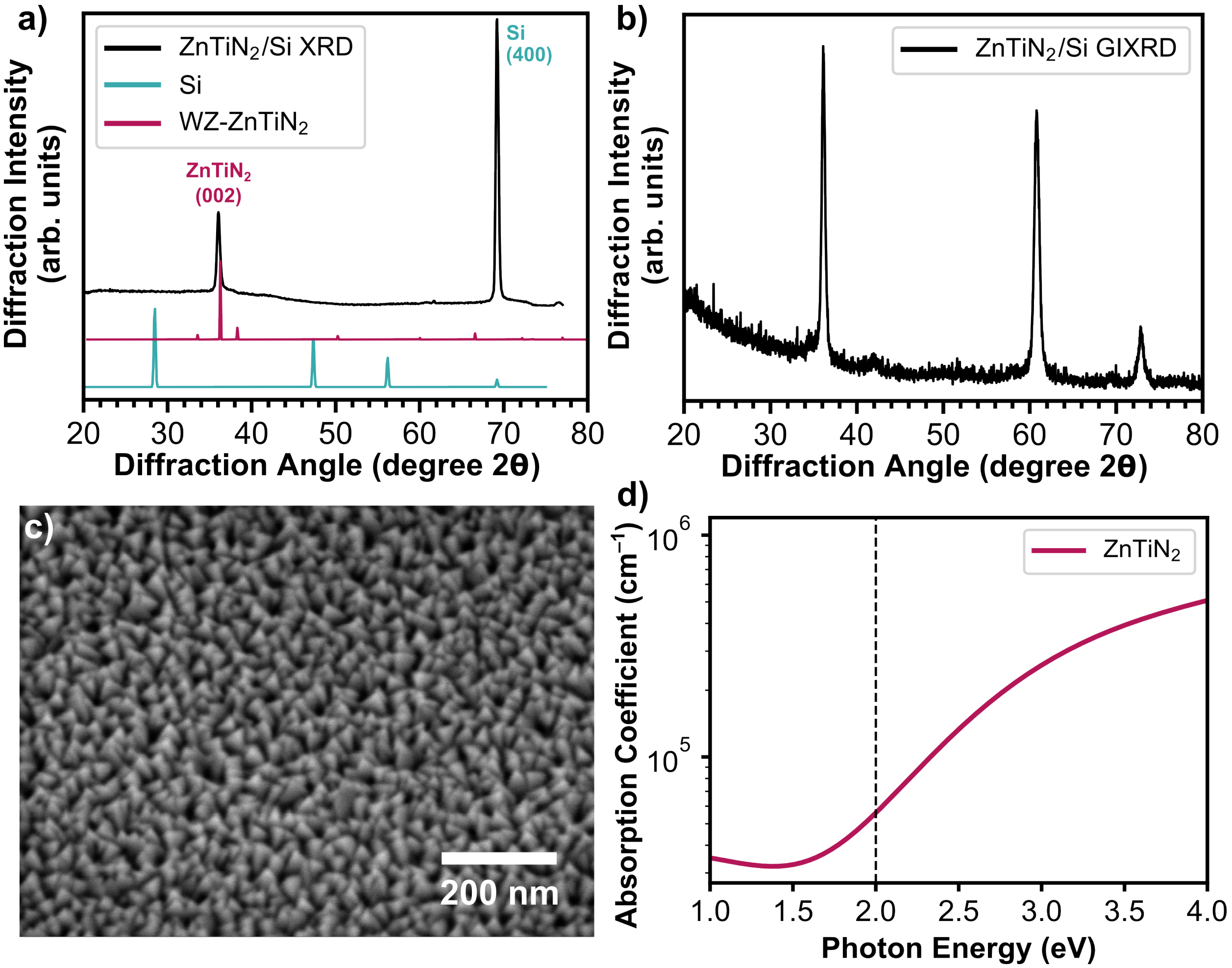

Figure S1. (a) X-ray diffraction (XRD) pattern of $ZnTiN_2$ film on Si (black) with reference spectra shown for Si (teal) and wurtzite $ZnTiN_2$ (magenta). (b) Surface-sensitive grazing incidence XRD (GIXRD) pattern of $ZnTiN_2$ film on Si. (c) Scanning electron micrograph of $ZnTiN_2$ film on Si surface tilted at 30 degrees to show texture. (d) Absorption coefficient vs incident photon energy for $ZnTiN_2$ film on Si, determined by spectroscopic ellipsometry. The dashed line corresponds to the approximate bandgap.

Physical and Optoelectronic Characterization of $ZnTiN_2$ film on Si

The single XRD peak at 2θ = 36° in **Figure S1a** is consistent with the (002) reflection of the wurtzite crystal structure typically adopted by cation-disordered $ZnTiN_2$ using our synthesis method, as we have shown in our previous works.[1,2] The prominence of this peak indicates a preferential growth in this direction, with the (002) planes oriented nominally parallel to the substrate growth surface. A silicon substrate peak is visible at 69°, corresponding to the (400) reflection (PDF file No. 01-070-5680), consistent with the (100) orientation of the wafer. GIXRD shows a similar peak at 2θ = 36° as well as additional major peaks and some minor peaks (**Figure S1b**). This indicates that the film was primarily (002) textured with some surface grains that are more polycrystalline or have a different preferred alignment. Nanocolumnar growth with

triangular faceting is seen in the scanning electron micrograph (**Figure S1c**). This morphology is typical of sputtered metal nitrides grown on non-templating substrates,[3–6] and is consistent with our previous work.[1,2] The absorption spectrum in **Figure S1d** was extracted by modeling the raw change in light polarization data with a PSemi-M0 oscillator and a Drude oscillator. The absorption onset around 1.8-2.0 eV indicates an optical bandgap in this range. Fitting the Drude oscillator to the sub-bandgap region revealed a charge carrier concentration of 2×10^{20} cm^{-3}, typical of novel ternary nitride materials under development.[7,8] Both of these results are consistent with our previous work.[1,2]

Current-Voltage Behavior of $ZnTiN_2$ Grown on n-GaN

$ZnTiN_2$ films were grown by radio-frequency co-sputtering on GaN substrates (GaN film grown by MOCVD on sapphire). To prepare samples as photoelectrodes, an ohmic contact to GaN was made using electron beam deposition of Ti/Al/Ni/Au, and conductive contacts and wire were electrically insulated from the electrolyte solution using epoxy and a glass tube. A bare n-type GaN substrate was used as a comparison photoelectrode. The current-voltage sweeps for GaN, a conductive carbon electrode, and $ZnTiN_2$/GaN in non-aqueous Fc are shown in **Figure S2**. Bare GaN acts as a photoanode, passing oxidizing current upon illumination. $ZnTiN_2$/GaN appears similar to the carbon electrode, with reducing and oxidizing current flowing regardless of illumination. This shows that $ZnTiN_2$ acts more like a conductor than a semiconductor, a result of its high dopant density.

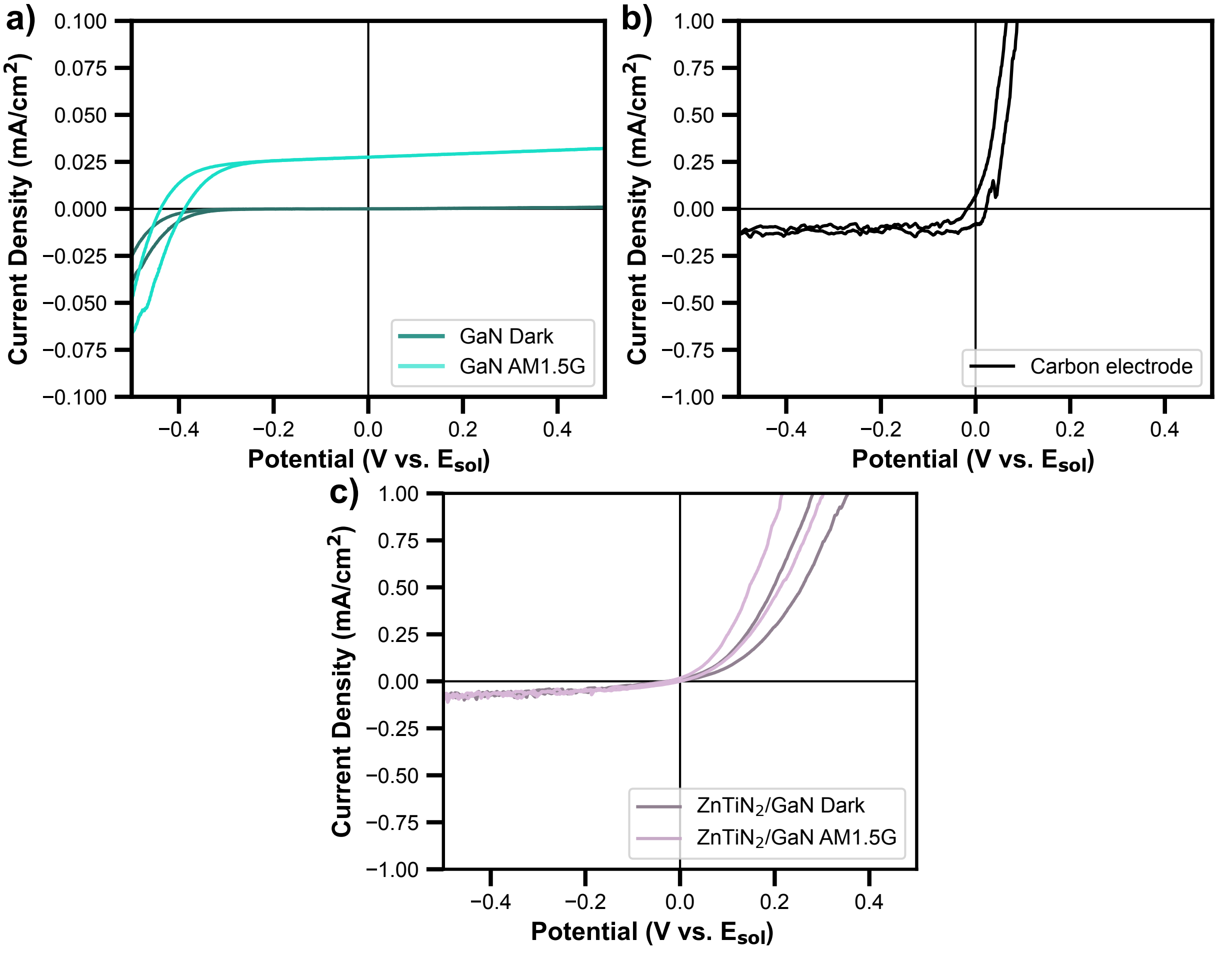

Figure S2. Photoelectrochemical characterization comparing current-voltage response of uncoated n-type GaN electrode and conductive carbon electrode to a $ZnTiN_2$-coated n-GaN electrode. (a) Cyclic voltammogram (CV) of GaN electrode in the dark and under simulated AM1.5G illumination, (b) CV of glassy carbon electrode, and (c) CV of $ZnTiN_2$/n-GaN electrode in the dark and under simulated AM1.5G illumination. Electrolyte: 0.5 M $TBAPF_6$, 0.01 M Fc^0, and 0.5 mM Fc^+ in dry acetonitrile. Light source was an LED-based solar simulator.

Determining the V_{OC} from Current-Voltage Curves of Si and $ZnTiN_2$/Si Samples

The method used to determine the V_{OC} from CVs of photoelectrodes is outlined in the methods section. Briefly, the point at which the current in the CV inflected downward was found to reasonably approximate the V_{OC} instead of where the current crossed zero.

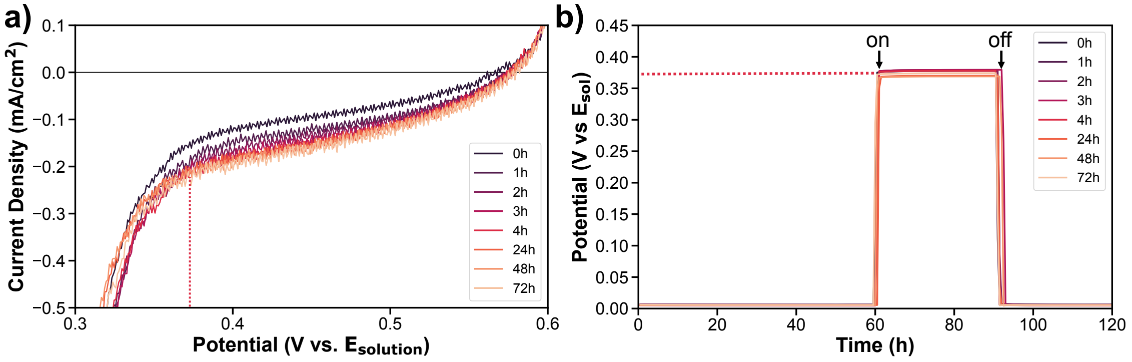

Figure S3. (a) Current-voltage curve of $ZnTiN_2$/Si framed to show the small cathodic current at low applied potential and the point at which the current crosses the x-axis. (b) Open circuit potential measurements at designated time points over 72 hours with the light turned on and off at the designated points. The vertical, red dashed line in (a) corresponds to the voltage of the horizontal, red dashed line in (b) to aid comparison of the data. Illumination was provided by an LED solar simulator.

Cross-sectional SEM to Corroborate Film Thickness

$ZnTiN_2$ film thickness on Si was found to be 190 nm by modeling of spectroscopic ellipsometry data. Cross-sectional SEM imaging corroborated this result, showing a film thickness of 180 nm (**Figure S4**).

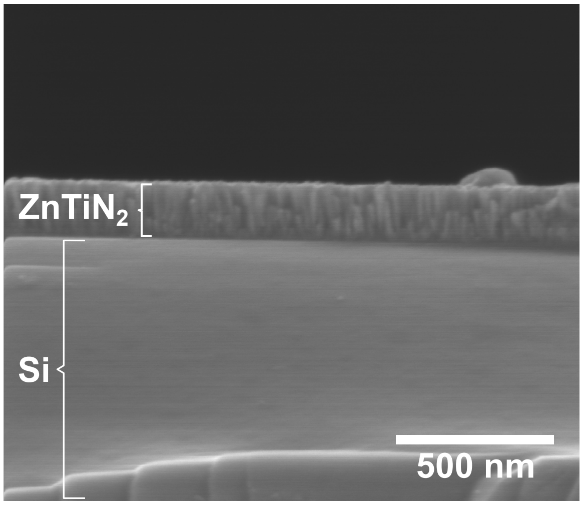

Figure S4. Scanning electron micrograph of the cross-section of $ZnTiN_2$ film on Si substrate to corroborate film thickness extracted from ellipsometry modeling. Accelerating voltage and current were 3 kV and 1.3 nA, respectively.

Incident Photon-to-Current Efficiency (IPCE) Measurement

$ZnTiN_2$/Si samples exhibited lower photocurrent densities compared to bare Si photocathodes, so IPCE was performed to assess the contribution of parasitic light absorption by the $ZnTiN_2$ film. As seen in **Figure S5**, the IPCE of $ZnTiN_2$/Si decreased around the bandgap of $ZnTiN_2$ identified in the absorption spectrum. These data confirm that Si was the active photoabsorber in $ZnTiN_2$/Si photocathodes and absorption by $ZnTiN_2$ did not add to the photocurrent, but rather decreased it. These results also confirm that despite high sub-bandgap absorption in the $ZnTiN_2$ layer, illumination successfully reaches the underlying Si to produce photocurrent.

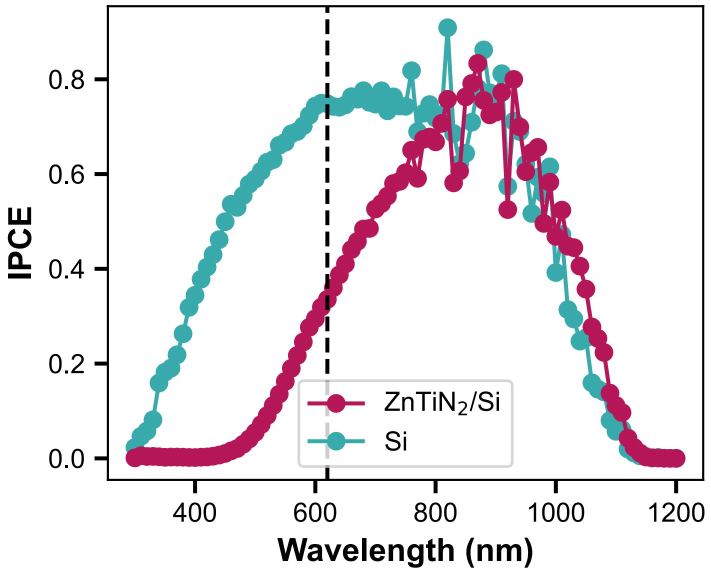

Figure S5. Incident photon to current efficiency measurements for (magenta) $ZnTiN_2$/Si and (teal) bare Si in 1 mM methyl viologen in pH 3.5 potassium hydrogen phthalate buffer at -0.35 V vs Ag/AgCl applied bias. The solution was not pre-electrolyzed before the IPCE experiment and thus only the oxidized half of the redox couple was present. Dashed black line indicates the approximate bandgap of $ZnTiN_2$ based on the absorption coefficient from spectroscopic ellipsometry. The light source was a Xe arc lamp passed through a monochromator. Spikes at wavelengths > 800 nm correspond to Xe emission lines and are the result of a non-linear relationship between the light intensity and the photocurrent response of the photoelectrodes.

Transient Reflectance (TR) Measurement

TR measurements were performed on Si and $ZnTiN_2$/Si to confirm that the film deposition process did not change the bulk carrier lifetime of the underlying Si (**Figure S6**). TR measurements were conducted with an excitation energy of 1.6 eV where a majority of photons are absorbed by the Si. For both samples, the excited state decays to the ground state with an 18 μs lifetime. The significant signal-to-noise ratio difference between the two samples may be due to the formation of an oxide layer in the Si only electrode, while the $ZnTiN_2$ may provide a protective coating effect to the Si preventing erosion of the TR signal.

The $ZnTiN_2$/Si TR spectra exhibit rapid evolution of ground state bleach features at 1.7 eV and 2.5 eV evolving to a photoinduced absorption (PIA) centered around 1.8 eV with small bleaches on either side. The long-lived PIA closely resembles that in the Si TR spectra. The initial changes in the $ZnTiN_2$/Si spectra may be due to remaining $ZnTiN_2$ excited state at early pump-probe delay times or an interaction between the $ZnTiN_2$ and Si at their interface. This evolution is complete within 1 μs and likely does not contribute significantly to the photoelectrochemical processes observed in **Figure 1**.

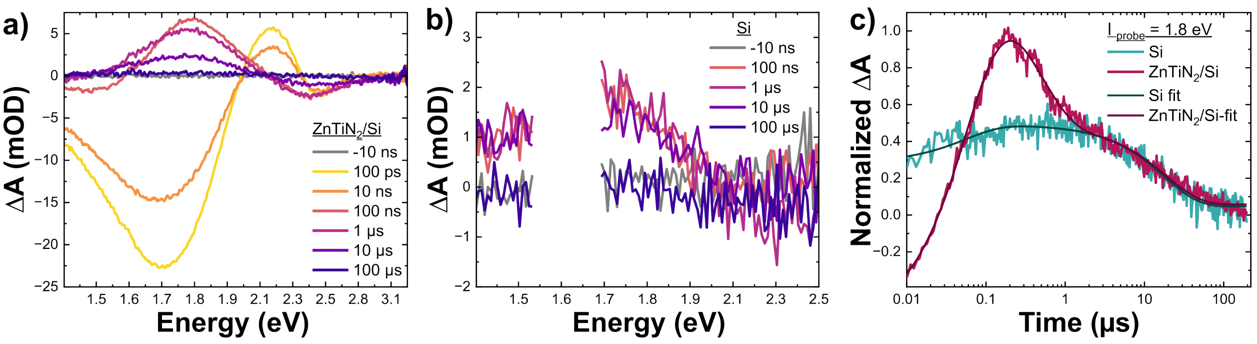

Figure S6. Time evolution of transient reflectance spectra taken for (a) $ZnTiN_2$/Si and (b) Si using an excitation energy of 1.6 eV. (c) Normalized change in absorbance of (magenta) $ZnTiN_2$/Si and (teal) Si at a probe energy of 1.8 eV.

"Deconstructable" Photoelectrode Preparation

To more easily characterize $ZnTiN_2$/Si samples after photoelectrochemical (PEC) treatment, a so-called "deconstructable" photoelectrode design was developed. As shown in **Figure S7**, electrodeposition tape was used as a mask to delineate the area that would be exposed to the electrolyte during photoelectrochemical testing. The same GaIn eutectic with Ag paste back contact was used on the unpolished side of the Si wafer. A flat wire coil was attached to the back and epoxied in place. A wire connection and glass tube could then be reversibly attached using electrodeposition tape and parafilm to protect the back contact from the electrolyte. This design allowed disassembly of the electrode to the flat geometry (Step 4 in **Figure S7**) for characterization by SEM, XPS, and AFM.

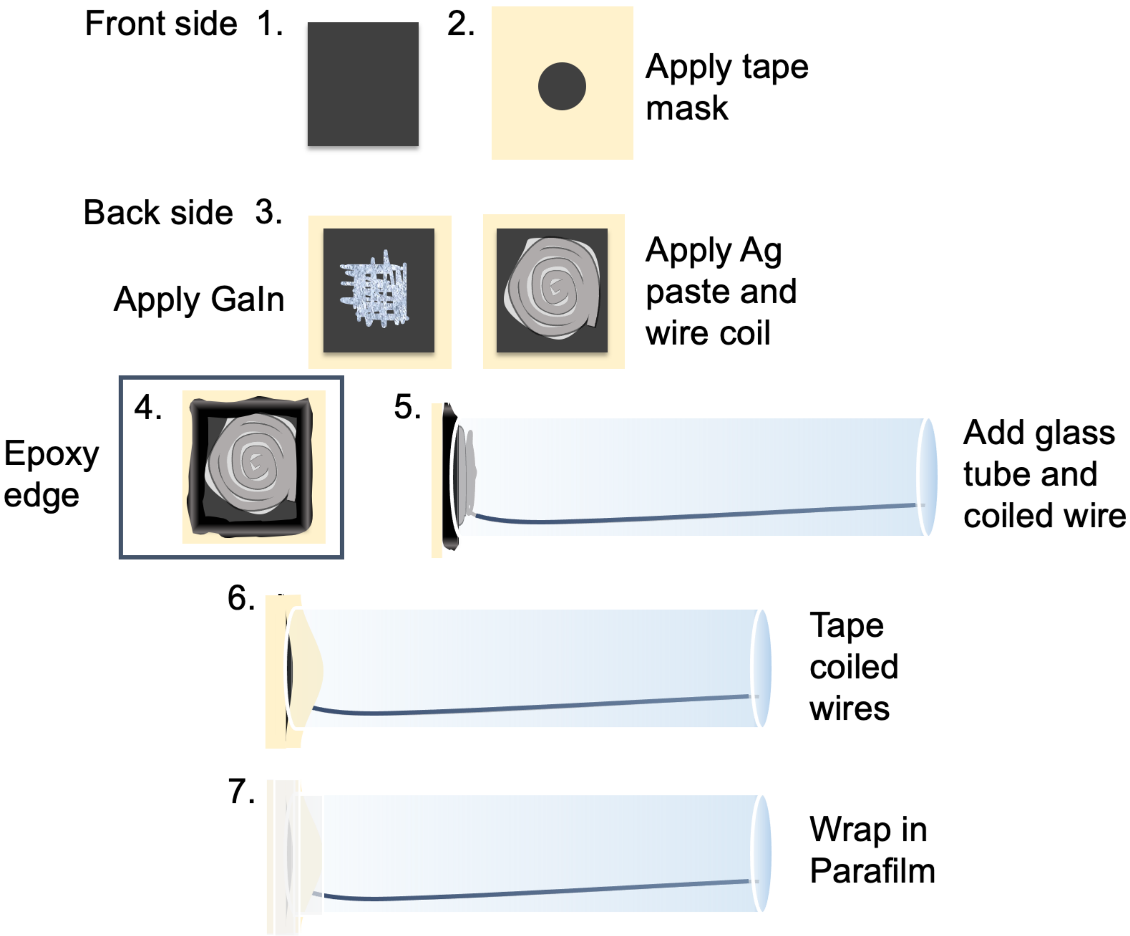

Figure S7. Schematic diagram of the process for fabricating "deconstructable" photoelectrodes for post-PEC characterization. Step 4 is outlined as the point the photoelectrode can be returned to as a flat geometry for characterization.

XPS Characterization of $ZnTiN_2$/Si

XPS was performed on the same $ZnTiN_2$/Si photoelectrodes as measured by EDS. XPS was also performed on a control $ZnTiN_2$/Si film that was not made into an electrode or exposed to electrolyte for comparison. The elemental composition data is provided in **Table S1**. XPS indicated that without electrolyte exposure, the $ZnTiN_2$ film surface was Zn-rich, while EDS and XRF showed that the bulk cation ratio was near 50:50. This was similarly observed in previous work.[1] After photoelectrochemical testing in pH 3.5 MV, the cation ratio (Zn/(Zn+Ti)) was lower compared to the control sample, consistent with the EDS data, the expectation from the Pourbaix diagram, and previous work.[1] After PEC testing in pH 9 MV, the cation ratio was similar compared to the control sample due to the high starting concentration of Zn in the control. O content was not higher in the samples exposed to electrolyte, likely because the control sample was exposed to air for long periods of time (days), resulting in a high surface O content.

Table S1. XPS elemental composition of all observed elements in $ZnTiN_2$/Si samples

	Zn/(Zn+Ti)	Zn	Ti	N	O	C	Si	Cl
**No electrolyte**	0.763	8.55	2.65	4.97	32.34	50.02	n.d.	1.47
**pH 3.5**	0.289	1.99	4.90	6.05	25.99	55.92	4.62	0.52
**pH 9**	0.837	6.64	1.29	4.11	23.04	54.98	3.95	5.99

XPS spectra for Zn, Ti, N, and O are provided in **Figure S8**. The results were generally consistent with findings in previous work.[1] The range of values displayed by the Zn 2p peaks (**Figure S8a**) were consistent with a Zn^{2+} oxidation state, which would be expected in ZnO and $ZnTiN_2$. The slight difference in position as a function of pH might relate to a more ZnO-like or $Zn(OH)_2$-like surface on the control and pH 9 samples and a more Zn-N-like contribution to the surface of the pH 3.5 sample.[9–11] This aligns with expectations from the Pourbaix diagram, as ZnO is not expected to be stable at pH 3.5.[1] The Ti 2p region (**Figure S8b**) clearly showed multiple peaks, indicating multiple chemical environments around Ti^{4+}, and the ratio of peak heights depended on sample treatment. We rule out a strong Ti^{3+} contribution, as the Ti^{3+} peak from TiN is well documented to be centered near 455 eV.[12–14] The lower energy peak (457 eV) was more prominent in the control sample, whereas the higher energy peak became more prominent after pH 3.5 electrolyte treatment. The high energy peak (458.5 eV) is consistent with the expectation for TiO_2, as is the appearance of a lower energy peak (530 eV) in the O 1s spectrum (**Figure S8d**),[15] providing further evidence for the formation of a TiO_2-like surface after operation in pH 3.5. The Ti 2p peaks became overall less prominent after exposure to pH 9 electrolyte, consistent with the loss of Ti from the surface. The N 1s peak around 396 eV (**Figure S8c**) could be consistent with either a nitride or an oxynitride phase.[9,12] The small peak at higher energy (400 eV) also suggests an oxynitride contribution, which might have become more oxidized after PEC operation at pH 9 to shift the peak to higher binding energy.[12,16] Particularly for the pH 9 sample, the change in the signal of the N 1s suggests that the changes to the $ZnTiN_2$ surface approach or exceed the detection depth of the XPS.

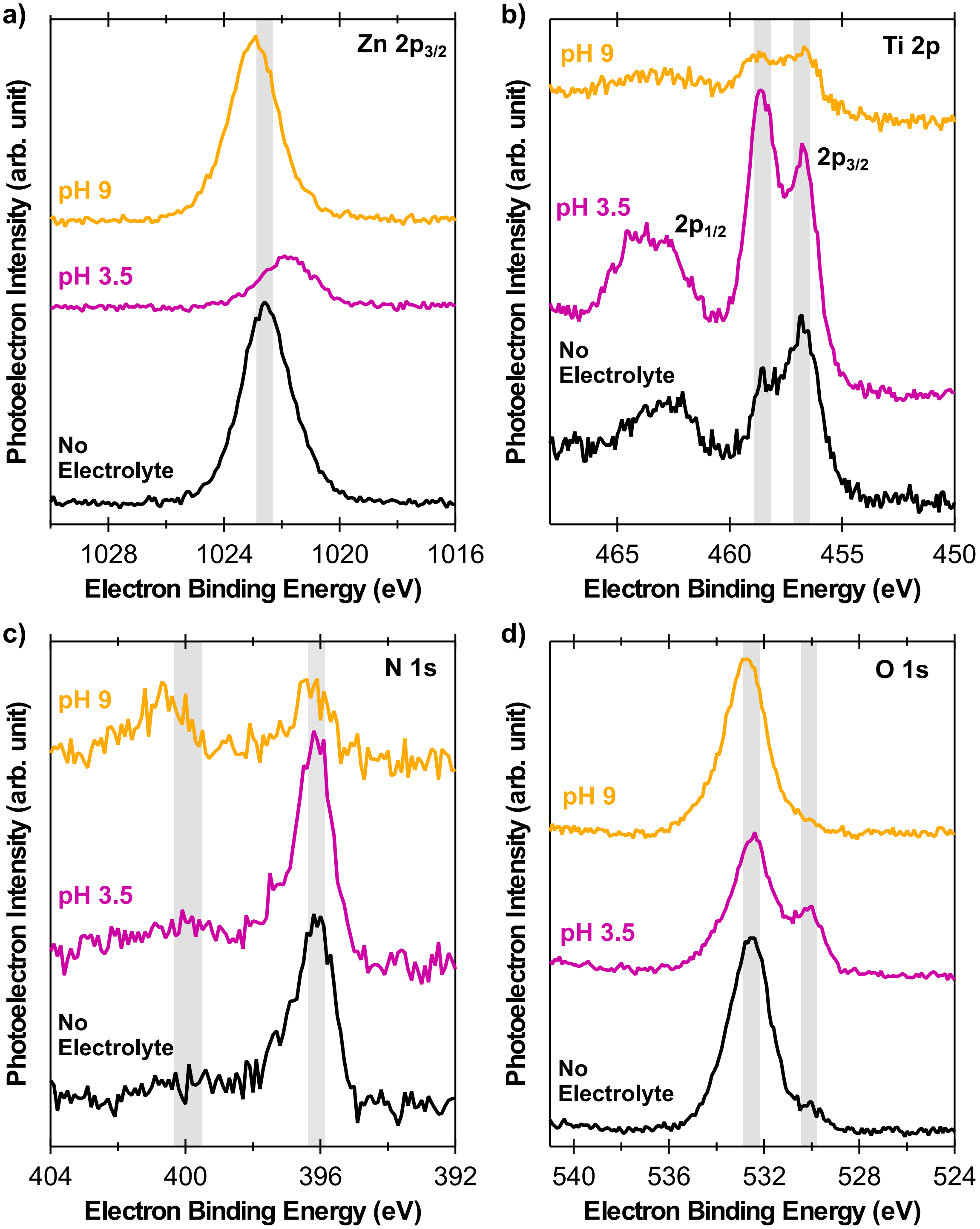

Figure S8. XPS spectra for elements (a) Zn, (b) Ti, (c) N, and (d) O in $ZnTiN_2$/Si photoelectrodes after photoelectrochemical operation in pH 3.5 (magenta) and pH 9 (orange) MV electrolyte. A film of $ZnTiN_2$/Si that was not made into an electrode or exposed to electrolyte was included as a negative control for comparison (black). The grey bars are a guide for the eye based on peak position in the control sample.

GIXRD of $ZnTiN_2$ Photoelectrodes after PEC Operation at pH 3.5 and pH 9

GIXRD was performed on the same photoelectrode samples that were measured by EDS and XPS. The diffraction patterns are shown in **Figure S9**. Analysis of the GIXRD data was carried out, but the patterns could not conclusively be indexed to a singular oxide phase, a mixture of known oxide phases, or to predicted ternary Zn-Ti-O phases. The zinc and titanium oxide, nitride or oxynitride phases that were investigated to index the GIXRD pattern were the following: rutile TiO_2 (ICSD 33837), anatase TiO_2 (ICSD 9852), brookite TiO_2 (ICSD 121631), wurtzite ZnO (ICSD 44477), cubic ZnO (ICSD 38222), rock salt TiN (ICSD 26947), hexagonal $Ti_3Zn_2O_8$ (Materials Project mp-1042495), cubic $Ti_3Zn_2O_8$ (Materials Project mp-29104), and $Ti_{2.85}O_4N$ (ICSD 173420). We also looked for peaks that may be due to the electrolyte salts including K_2SO_4. None of the phases or mixtures of phases included could provide a comprehensive fit of the locations and relative intensities of the diffraction peaks in the GIXRD. However, despite inconclusive phase identification, the results show that many additional peaks became visible after PEC operation in both pH conditions compared to the $ZnTiN_2$/Si film that was not made into a photoelectrode; the peak positions, relative intensities, and number of peaks were different depending on the pH of the electrolyte the $ZnTiN_2$/Si was exposed to; and the peaks observed in the film that was not made into an electrode are almost completely suppressed in the photoelectrode samples. This shows that the surface chemistries became different as a result of exposure to aqueous electrolyte and the specific surface chemistry was dependent on the pH of the electrolyte, as expected from the Pourbaix diagram.[1] These changes to the surface chemistry are considerable, given that the oxide/mixed phase crystallites coherently diffract. However, the peaks are not identifiable to specific phases due to the multitude of possible Zn-Ti-O-N phases, overlapping peaks, and peak shifting due to ill-defined atom positioning within the crystal lattice (e.g., the cations or the anions swapping sites).

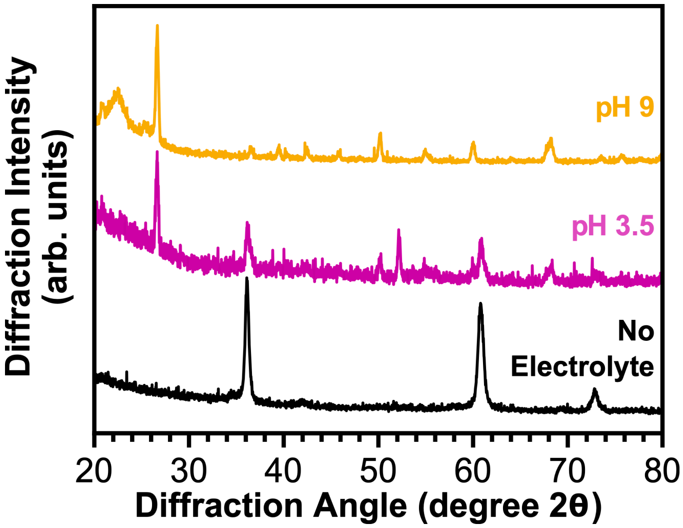

Figure S9. GIXRD patterns for $ZnTiN_2$/Si photoelectrodes after PEC operation at pH 3.5 (pink) and pH 9 (yellow). GIXRD pattern for $ZnTiN_2$/Si film that was not made into an electrode or exposed to electrolyte (black) is the same as shown in **Figure S1b**.

Energy Dispersive X-Ray Spectroscopy (EDS) of deconstructable electrodes

EDS was used to measure the elemental abundance of Zn, Ti, N, and O in areas of the same electrode that were either unexposed or exposed to electrolyte due to masking with

electrodeposition tape. Deconstructable electrodes (fabrication scheme shown in **Figure S7**) were subjected to 1 h at open circuit in the dark and 1 h at short circuit under simulated sunlight. The EDS measurements taken with a 10 kV accelerating voltage (**Figure S10**) agree with the measurements taken using a 3 kV accelerating voltage (**Figure 5c**), showing a decrease in Zn content in areas exposed to pH 3.5 electrolyte/PEC operation and an increase in O content in areas exposed to electrolyte of either pH. In these plots, the Zn, Ti, N, and O are normalized to the sum of these elements. **Table S2** shows the full EDS values summed to the total of all elements detected.

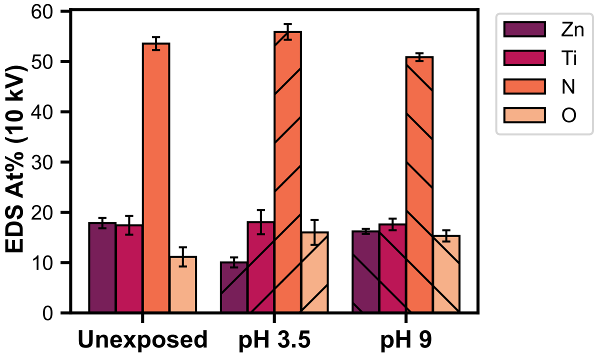

Figure S10. Bar plot depicting the bulk elemental abundance of Zn, Ti, N, and O by EDS on $ZnTiN_2$ photoelectrodes in areas unexposed to electrolyte (control; left) and exposed to electrolyte for PEC operation in pH 3.5 (middle) and pH 9 (right) MV. Data were taken using an accelerating voltage of 10 kV and beam current of 2.6 nA.

Table S2. EDS elemental composition of all observed elements in $ZnTiN_2$/Si samples in areas unexposed and exposed to electrolyte

**3 kV**								
		**Zn/(Zn+Ti)**	**Zn**	**Ti**	**N**	**O**	**C**	**Si**
**pH 3.5**	**Unexposed**	0.49	17.89	18.91	36.26	9.88	16.25	0.81
	**Exposed**	0.38	12.30	19.73	33.01	13.81	20.24	0.90
**pH 9**	**Unexposed**	0.49	17.98	18.45	34.27	10.31	18.49	0.50
	**Exposed**	0.51	18.36	17.68	33.51	16.44	12.71	1.30
**10 kV**								
**pH 3.5**	**Unexposed**	0.49	7.95	8.22	25.70	5.63	23.02	29.48
	**Exposed**	0.36	4.29	7.71	23.87	6.84	21.65	35.64
**pH 9**	**Unexposed**	0.51	9.33	9.10	27.97	5.82	19.93	27.85
	**Exposed**	0.48	8.69	9.43	27.26	8.21	15.85	30.56

Note on EDS quantification of Si on $ZnTiN_2$/Si electrodes in unexposed vs exposed areas

We observed a slight tendency for the exposed areas of samples to have a higher atomic percentage of Si by EDS (**Table S3**). This included a $ZnTiN_2$/Si electrode that was prepared with a tape mask but not exposed to electrolyte (air exposed), but the difference was slightly larger in samples exposed to electrolyte. This higher Si signal might suggest that the $ZnTiN_2$ layer was thinning. Measurements after more prolonged PEC operation would be needed to increase the certainty of this interpretation.

Table S3. EDS atomic percent of Si in $ZnTiN_2$/Si samples

	**3 kV**		**10 kV**	
**Treatment**	**Unexposed**	**Exposed**	**Unexposed**	**Exposed**
**pH 3.5**	0.81	0.90	29.48	35.64
**pH 9**	0.50	1.30	27.85	30.56
**Air Exposed**	0.32	0.11	23.61	25.40

Atomic force microscopy (AFM) roughness of $ZnTiN_2$/Si after exposure to electrolyte

AFM was performed on $ZnTiN_2$/Si electrodes that were operated in pH 3.5 and pH 9 MV (described previously) and on a $ZnTiN_2$/Si film that was not made into an electrode or exposed to electrolyte as a control. **Table S4** provides the average root mean squared (RMS) roughness, and representative AFM images are shown in **Figure S11**. There was no significant change in the RMS roughness after operation in pH 3.5 or pH 9 MV for 1 h in the dark at open circuit and 1 h under illumination at short circuit. This either indicates that the films were robust to degradation during PEC operation or that film degradation occurred uniformly across the surface topography, leaving surface features intact.

Table S4. AFM RMS roughness of $ZnTiN_2$/Si samples without and with electrolyte exposure

**Treatment**	**Roughness (nm)**
**No electrolyte**	5.32
**pH 3.5**	6.07
**pH 9**	5.14

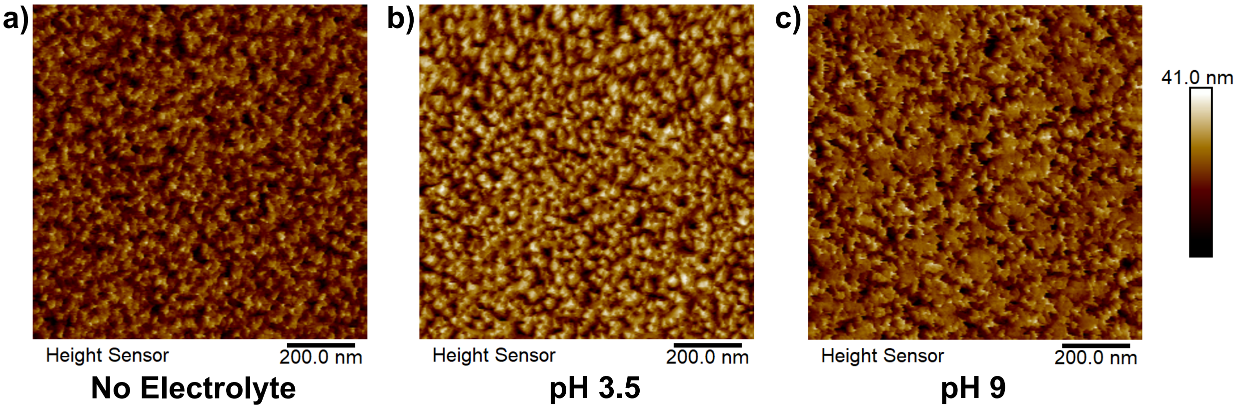

Figure S11. (a) Atomic force microscopy (AFM) topography of $ZnTiN_2$/Si film that was not made into an electrode or exposed to electrolyte. (b) and (c) AFM topography of $ZnTiN_2$/Si electrodes after exposure to electrolyte for PEC operation in pH 3.5 and pH 9, respectively. The scan rate was 0.702 Hz in (a) and 1.50 Hz in (b) and (c). The color scale applies to all three samples.

References

(1) Greenaway, A. L.; Ke, S.; Culman, T.; Talley, K. R.; Mangum, J. S.; Heinselman, K. N.; Kingsbury, R. S.; Smaha, R. W.; Gish, M. K.; Miller, E. M.; Persson, K. A.; Gregoire, J. M.; Bauers, S. R.; Neaton, J. B.; Tamboli, A. C.; Zakutayev, A. Zinc Titanium Nitride

Semiconductor toward Durable Photoelectrochemical Applications. *J. Am. Chem. Soc.* **2022**, *144* (30), 13673–13687. https://doi.org/10.1021/jacs.2c04241.

(2) Mangum, J. S.; Ke, S.; Gish, M. K.; Raulerson, E. K.; Perkins, C. L.; Neaton, J. B.; Zakutayev, A.; Greenaway, A. L. Sn-Assisted Heteroepitaxy Improves $ZnTiN_2$ Photoabsorbers. *J. Mater. Chem. A* **2024**, *12* (8), 4544–4554. https://doi.org/10.1039/D3TA06200G.

(3) Mahieu, S.; Buyle, G.; Ghekiere, P.; Heirwegh, S.; Gryse, R. D.; Depla, D. Mechanism of Biaxial Alignment in Thin Films, Deposited by Magnetron Sputtering. *Thin Solid Films* **2006**, *515* (2), 416–420.

(4) Mahieu, S.; Ghekiere, P.; Depla, D.; Gryse, R. D. Biaxial Alignment in Sputter Deposited Thin Films. *Thin Solid Films* **2006**, *515* (4), 1229–1249.

(5) Chinsakolthanakorn, S. Characterization of Nanostructured TiZrN Thin Films Deposited by Reactive DC Magnetron Co-Sputtering. *Procedia Eng.* **2012**.

(6) Islam, M. Stable Stoichiometric Copper Nitride Thin Films via Reactive Sputtering. *Appl. Phys. A* **2022**, *128*. https://doi.org/10.1007/s00339-022-05726-3.

(7) Melamed, C. L.; Miller, M. K.; Cordell, J.; Pucurimay, L.; Livingood, A.; Schnepf, R. R.; Pan, J.; Heinselman, K. N.; Vila, F. D.; Mis, A.; Nordlund, D.; Levy-Wendt, B.; Lany, S.; Toberer, E. S.; Christensen, S. T.; Tamboli, A. C. Short-Range Order Tunes Optical Properties in Long-Range Disordered $ZnSnN_2$–ZnO Alloy. *Chem. Mater.* **2022**, *34* (9), 3910–3919. https://doi.org/10.1021/acs.chemmater.1c03938.

(8) Greenaway, A. L.; Loutris, A. L.; Heinselman, K. N.; Melamed, C. L.; Schnepf, R. R.; Tellekamp, M. B.; Woods-Robinson, R.; Sherbondy, R.; Bardgett, D.; Bauers, S.; Zakutayev, A.; Christensen, S. T.; Lany, S.; Tamboli, A. C. Combinatorial Synthesis of Magnesium Tin Nitride Semiconductors. *J. Am. Chem. Soc.* **2020**, *142* (18), 8421–8430. https://doi.org/10.1021/jacs.0c02092.

(9) Zhuk, S.; Siol, S. Chemical State Analysis of Reactively Sputtered Zinc Vanadium Nitride: The Auger Parameter as a Tool in Materials Design. *Appl. Surf. Sci.* **2022**, *601*, 154172. https://doi.org/10.1016/j.apsusc.2022.154172.

(10) He, B.; Yuan, Y.; Wang, J.; Pervaiz, E.; Dong, X.; Shao, Z.; Yang, M. Hierarchical Ni_3ZnN Hollow Microspheres as Stable Non-Noble Metal Electrocatalysts for Oxygen Reduction Reactions. *Electrocatalysis* **2018**, *9* (4), 452–458. https://doi.org/10.1007/s12678-018-0461-7.

(11) National Institute of Standards and Technology. NIST X-Ray Photoelectron Spectroscopy Database, NIST Standard Reference Database Number 20, 2023. https://dx.doi.org/10.18434/T4T88K.

(12) Saha, N. C.; Tompkins, H. G. Titanium Nitride Oxidation Chemistry: An X-Ray Photoelectron Spectroscopy Study. *J. Appl. Phys.* **1992**, *72* (7), 3072–3079. https://doi.org/10.1063/1.351465.

(13) Glaser, A.; Surnev, S.; Netzer, F. P.; Fateh, N.; Fontalvo, G. A.; Mitterer, C. Oxidation of Vanadium Nitride and Titanium Nitride Coatings. *Surf. Sci.* **2007**, *601* (4), 1153–1159. https://doi.org/10.1016/j.susc.2006.12.010.

(14) Esaka, F.; Furuya, K.; Shimada, H.; Imamura, M.; Matsubayashi, N.; Sato, H.; Nishijima, A.; Kawana, A.; Ichimura, H.; Kikuchi, T. Comparison of Surface Oxidation of Titanium Nitride and Chromium Nitride Films Studied by X-Ray Absorption and Photoelectron Spectroscopy. *J. Vac. Sci. Technol. Vac. Surf. Films* **1997**, *15* (5), 2521–2528. https://doi.org/10.1116/1.580764.

(15) Diebold, U.; Madey, T. E. TiO_2 by XPS. *Surf. Sci. Spectra* **1996**, *4* (3), 227–231. https://doi.org/10.1116/1.1247794.
(16) Milošev, I.; Strehblow, H.-H.; Navinšek, B. Oxidation of Ternary TiZrN Hard Coatings Studied by XPS. *Surf. Interface Anal.* **1998**, *26* (4), 242–248. https://doi.org/10.1002/(SICI)1096-9918(199804)26:4<242::AID-SIA367>3.0.CO;2-Y.
